# A novel free-operant framework enables experimental habit induction in humans

**DOI:** 10.3758/s13428-023-02263-6

**Published:** 2023-11-21

**Authors:** Rani Gera, Segev Barak, Tom Schonberg

**Affiliations:** 1 Sagol School of Neuroscience, Tel Aviv University, Tel Aviv, Israel; 2 School of Psychological Sciences, Tel Aviv University, Tel Aviv, Israel; 3 School of Neurobiology, Biochemistry and Biophysics, The George S. Wise Faculty of Life Sciences, Tel Aviv University, Tel Aviv, Israel; 4 Division of Humanities and Social Sciences, California Institute of Technology, Pasadena, CA USA

**Keywords:** Habits, Goal-directed behavior, Motivation, Model-based learning, Model-free learning, Free-operant, Real-world, Mobile application, Reward, Learning

## Abstract

Habits are a prominent feature of both adaptive and maladaptive behavior. Yet, despite substantial research efforts, there are currently no well-established experimental procedures for habit induction in humans. It is likely that laboratory experimental settings, as well as the session-based structure typically used in controlled experiments (also outside the lab), impose serious constraints on studying habits and other effects that are sensitive to context, motivation, and training duration and frequency. To overcome these challenges, we devised a unique real-world free-operant task structure, implemented through a novel smartphone application, whereby participants could freely enter the app (24 hours a day, 7 days a week) to win rewards. This procedure is free of typical laboratory constraints, yet well controlled. Using the canonical sensitivity to outcome devaluation criterion, we successfully demonstrated habit formation as a function of training duration, a long-standing challenge in the field. Additionally, we show a positive relationship between multiple facets of engagement/motivation and goal-directedness. We suggest that our novel paradigm can be used to study the neurobehavioral and psychological mechanism underlying habits in humans. Moreover, the real-world free-operant framework can potentially be used to examine other instrumental behavior-related questions, with greater face validity in naturalistic conditions.

**Supplementary Information:**

The online version contains supplementary material available at 10.3758/s13428-023-02263-6.

## Introduction

Habits are a fundamental feature of both adaptive and maladaptive human behavior. According to the dual process theory of action control (De Wit & Dickinson, 2009; Dickinson, 1985), newly learned behaviors, acquired through the process of instrumental learning, are at first goal-directed. Goal-directed action control is thought to rely mainly on the association between a response and its outcome (Balleine & O’Doherty, 2010; Dickinson, 1985), so it can be flexibly modified or temporarily suspended (e.g., Valentin et al., 2007). This flexibility comes with the cost of substantial cognitive resources utilized for the consideration and planning of the actions. Following extensive training in similar conditions, actions are likely to become predominantly habitual, liberating valuable cognitive resources while allowing patterns of actions to be performed effortlessly (Balleine & O’Doherty, 2010; Dickinson, 1985; Graybiel, 2008). Habitual actions are thought to rely mainly on the association between a cue and a response, where the cue elicits an automatic response (Adams, 1982; Dickinson et al., 1995). Therefore, behaviors under habitual action control are relatively fixed and cannot be easily adjusted when circumstances advantage a different pattern of action.

While primarily adaptive, the habitual system is also crucially involved in the manifestation of maladaptive, unwanted behaviors, making their elimination particularly difficult. Specifically, an imbalance between the goal-directed and habitual systems—and particularly, overreliance on habitual action control—has been implicated in a variety of prevalent psychopathologies, including addiction (Ersche et al., 2016; Hogarth et al., 2012; Hogarth & Chase, 2011; McKim et al., 2016; Sjoerds et al., 2013), obsessive–compulsive disorder (Gillan et al., 2011, 2014; Gillan, Apergis-Schoute, et al., 2015a; Snorrason et al., 2016), social anxiety (Alvares et al., 2014, 2016), and obesity (Horstmann et al., 2015). Therefore, characterizing habit formation and manifestation, and understanding the interplay between goal-directed and habitual action control are of great clinical and societal importance.

The gold-standard criterion for distinguishing habitual from goal-directed action control has been the sensitivity of a learned behavior to changes in its outcome value (Adams, 1982; Dickinson, 1985). Goal-directed behavior is sensitive to changes in outcome value, whereas habits are insensitive to such changes. This criterion was utilized in a landmark rodent study (Adams, 1982) that tested changes in response rates towards a food reinforcer following its devaluation (using conditioned taste aversion or, in later research, food satiety; Balleine & Dickinson, 1998). A particularly important demonstration of this work has been the shift from goal-directed to habitual action control as a function of training duration (Adams, 1982), which is arguably the major route through which habits are established. This result led to studies that yielded a substantial body of knowledge on habits in animals (Balleine, 2019; Balleine & O’Doherty, 2010).

Habit research in humans has utilized the outcome devaluation criterion to point to differences in habit expression in neuropsychiatric disorders (Alvares et al., 2016; Gillan et al., 2014; Hogarth et al., 2012; Luijten et al., 2020) and search for relevant psychological factors and individual characteristics that modulate habit expression (Gillan, Otto, et al., 2015b; Pool et al., 2022; Schwabe & Wolf, 2009). It has also been used in attempts to dissociate the neural correlates of the goal-directed and habitual systems (e.g., de Wit et al., 2012; Liljeholm et al., 2015; Reber et al., 2017; Valentin et al., 2007; Watson et al., 2018). However, perhaps the most basic demonstration of habit formation through prolonged training has yet to be reliably demonstrated. Only a single study has successfully demonstrated the shift in action control as a function of training duration (Tricomi et al., 2009) but those results have not been replicated thus far and have been challenged in multiple replication attempts (de Wit et al., 2018; Gera et al., 2022; Pool et al., 2022). This impedes our ability to understand the mechanisms underlying habit formation and the development of potential interventions, and has left the research of habit formation in humans far behind its animal counterpart.

Here, we addressed the fundamental need for a habit induction task in humans (based on training duration). We rationalized that the discrepancy between animal and human research in the field of habit formation has been largely driven by laboratory limitations in human experimental settings. First, habits are highly dependent on context (Bouton, 2021; Thrailkill & Bouton, 2015), and laboratory settings in human experiments are different from common daily contexts. Second, most laboratory experiments in humans offer a relatively short duration of training, whereas real-life habits are established over a substantial period of time (Lally et al., 2010), ranging from weeks to months. This long timeframe is feasible for animal research but not for human laboratory research. Relatedly, laboratory experiments in humans typically involve massed training, whereas habit formation favors distributed training (Adams, 1982). A recent study provided compelling evidence supporting the significant impact of training density on human reinforcement-based learning (Wimmer et al., 2018). The study elucidated distinct cognitive and neural mechanisms engaged in reinforcement-based learning under massed versus spaced training and indicated better sustainability of associations learned through the latter regimen. Finally, in instrumental training in laboratory settings, an unnatural situation is imposed on participants. In both free-operant and trial-based tasks, participants are “forced” to engage with a task for a substantial amount of time with a very narrow range of possible actions and, importantly, without the opportunity to stop engaging with the task in favor of another activity. This undermines the motivational aspects of their behavior (which play a dominant role in the process of habit formation) and is remote from how habits are typically acquired in real life.

**Fig. 1:**
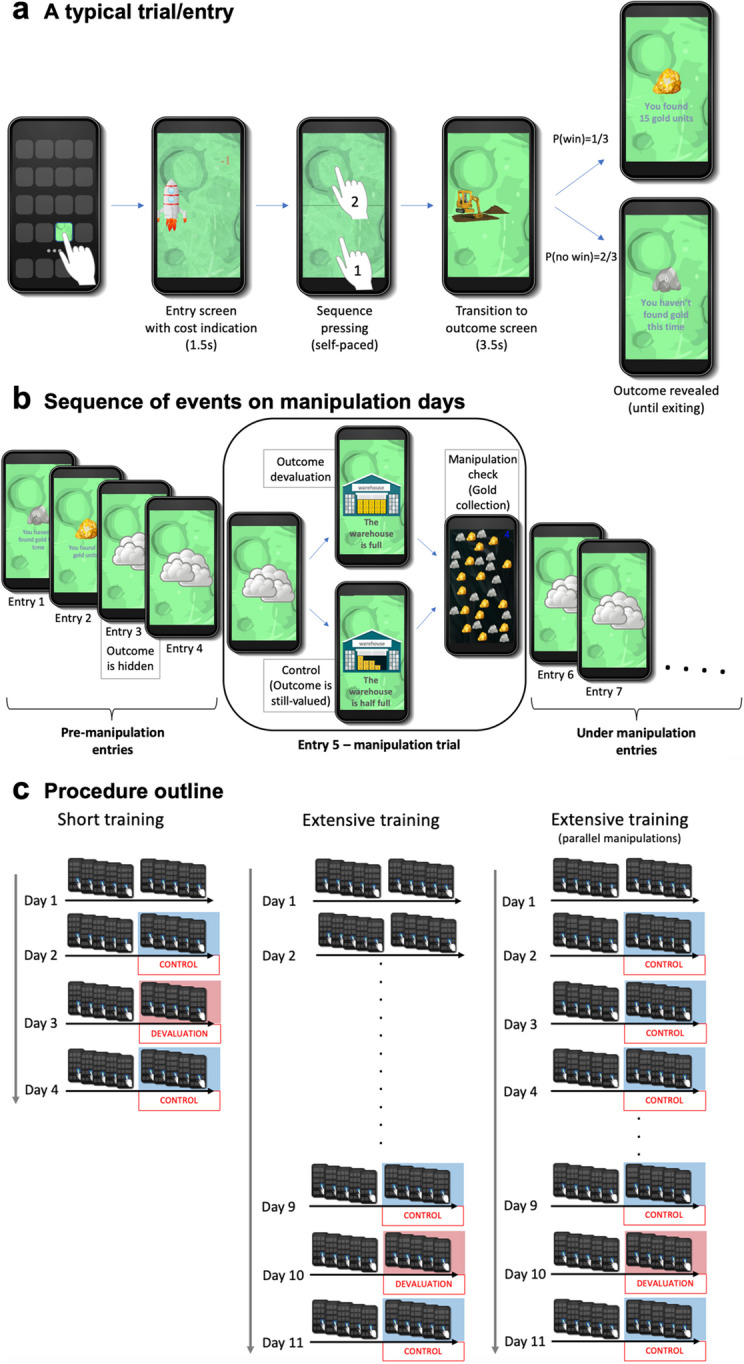
**Illustration of the experimental procedure.** During the experiment, participants were free to enter a gamified experimental app to find gold (converted into real money in case a dedicated warehouse is not full) on a distant planet (“the gold planet”). Participants were free to enter whenever and as much as they liked (24/7). (**a**) On a typical entry/trial, participants first viewed an animation of their spaceship landing on the gold planet and an indication of the cost each entry involved (one unit of gold). Then, to start looking for gold, they had to press the lower and upper half of the screen (in this fixed order), followed by a digging for gold animation that ended with the outcome presentation (either 15 units of gold or a worthless piece of rock). (**b**) On manipulation days (unbeknownst to participants), the day started regularly. From the third daily entry and until the next day, the outcome was hidden. On the fifth entry, the participants were presented with either a message stating that the warehouse had reached capacity, effectively meaning they could not accumulate any more of the gold they would possibly find for the rest of the day (outcome devaluation), or a message stating that the warehouse had become half full, meaning they could continue to accumulate gold (a control manipulation). After confirming this message, they found a cave rich with gold and had 5 seconds to collect gold (by pressing the gold piles). Each press in the cave cost 10 gold units, and each pile was worth 15 units, which could only be accumulated if the warehouse was not full. This part was used as a manipulation check for the outcome devaluation. Subsequent entries during the rest of the day were considered entries under manipulation and were used as the main dependent variable. (**c**) Participants were assigned to three experimental groups that varied in training duration and number of control manipulations. * An online demo of the task is accessible at https://ranigera.github.io/RWFO_app_demo

To overcome these challenges, we developed a novel smartphone application (to be denoted as “app”), installed on participants’ own mobile devices. The app implements a gamified real-world free-operant task in which participants are allowed to freely and voluntarily determine their pattern of engagement (while they go on with their everyday lives). In our task, participants were instructed that they could enter the app whenever and as much as they liked 24 hours a day, 7 days a week, in order to land their spaceship on a planet rich with gold and remove an ice layer (by pressing a short sequence on the screen) to search for gold (worth real money; Fig. 1). A typical entry lasted ~8–10 seconds, and participants found gold every three entries on average. We informed participants that the gold they found was stored in a warehouse that could be filled to capacity. Participants were assigned to three experimental groups that underwent either short (one group) or extensive training (two groups). On the third day (short training group) or tenth day (extensive training groups), the warehouse had become full, thereby preventing further gold accumulation. This served as the outcome devaluation manipulation. Notably, each entry to the app had a small cost, set to prevent intentional entries following outcome devaluation (Gillan, Otto, et al., 2015b). The warehouse was emptied at the beginning of each day, thus allowing repeated manipulations. To obtain a within-participant baseline measurement, we deployed a control manipulation (where the warehouse became half full) a day before and a day after the outcome devaluation day. One of the two extensive training groups performed an additional three control manipulations on the second, third, and fourth days (to occur chronologically in parallel days to the days of manipulations in the short training group). We sought to test whether and to what extent participants continued to enter the app following outcome devaluation compared with the preceding and following adjacent “outcome value retained” days (devaluation day ±1). We hypothesized that our procedure would lead to increased habit formation as a function of training duration, such that participants would demonstrate reduced sensitivity to outcome devaluation following extensive (long) training compared with short training. As an outcome devaluation manipulation check, we embedded a mini-task following each manipulation during which participants had 5 seconds to freely collect gold at a substantial cost. An online demo of the task is accessible at https://ranigera.github.io/RWFO_app_demo. We also examined whether habit expression was associated with measures of engagement/motivation (extracted from the task data) and with the reliance on model-free (MF) and model-based (MB) reinforcement learning strategies—as extracted from the commonly used two-step task (Daw et al., 2011) that participants performed following the main task—which are often referred to as proxies of habitual and goal-directed behavior, respectively.

## Results

### General engagement rates throughout the task

**Fig. 2:**
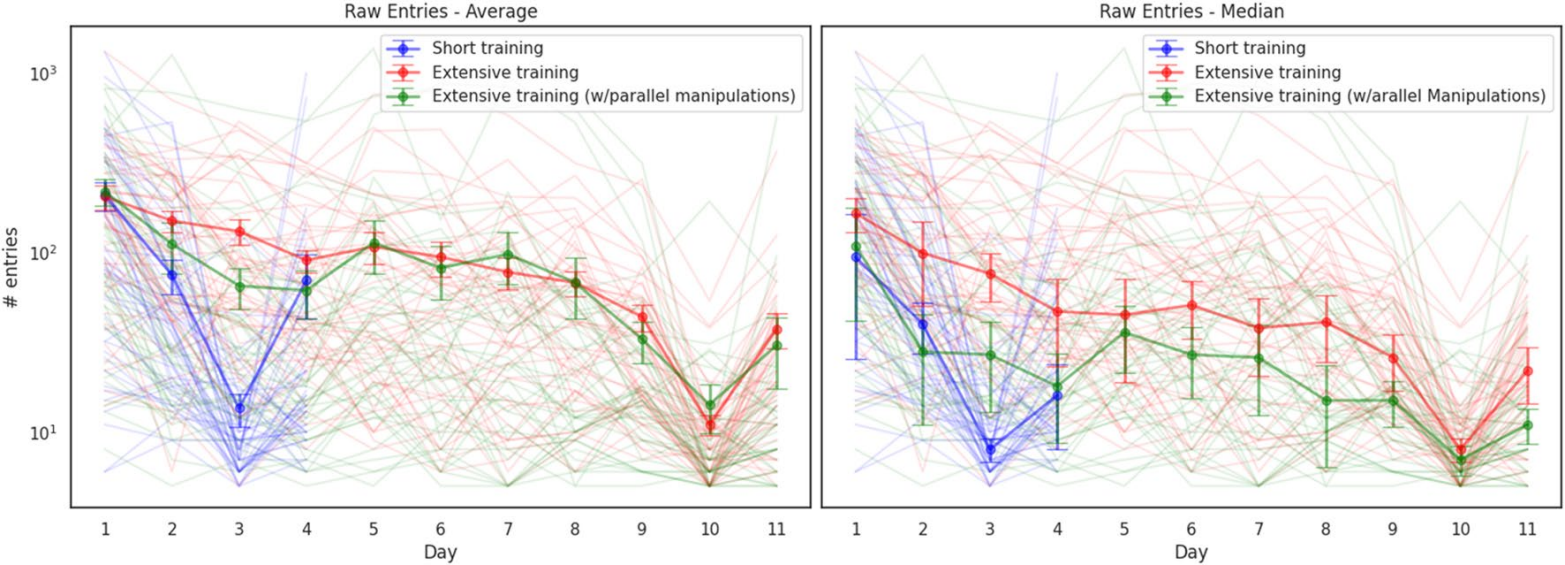
**Raw daily entry data.** Average (on the left panel) and median (on the right panel) numbers of entries to the app on each experimental day. Error bars on the left panel represent ±1 standard error of the mean (SEM) and on the right panel represent estimated 95% CI around the median (calculated as $Median\pm \frac{1.57\ x\ IQR}{\surd N}$, similar to boxplot notches). Semitransparent lines represent individual participants

Engagement rates throughout the task varied quite considerably between participants. Across all participants and all days of the task, excluding manipulation days, participants entered an average of 122.2 entries a day (*SD* = 180.7; median = 53.5, estimated 95% CI around the median [46.9, 61.1]; Fig. 2 and Supplementary Fig. 1).

### Habit formation as a function of training duration

To test the effect of training duration on habit formation, we ran our preregistered mixed-model Poisson regression with number of entries as the dependent variable, group (short training, extensive training, and extensive training with parallel manipulations) and manipulation (outcome devaluation, control pre-devaluation, control post-devaluation) and their interaction, as independent variables, and participant as a random effect (see formulation and a required slight deviation from our preregistration in “Data analysis”). The results of the model showed a large over-dispersion (dispersion ratio = 11.683, Pearson’s χ^2^_389_ = 4545, *p* < 0.001), indicating that this model is not suitable for our data. In search of an appropriate model, we ran a leave-one-out cross-validation (LOOCV) on three models proposed to adequately handle over-dispersed count data (Bolker, 2022): an observation-level random effects model (OLRE), a negative binomial mixed-model regression with a linear (“quasi-Poisson”) parameterization (NB1), and a similar model with a quadric parameterization of the variance (NB2). All models were formulated identically as the aforementioned mixed-model Poisson regression, with the exception of the observation-level random effect added in the OLRE model. The NB1 model yielded the lowest mean squared error (MSE) and thus was chosen to model our data (for the results of the unchosen models see Supplementary Tables 1 and 2).

**Fig. 3:**
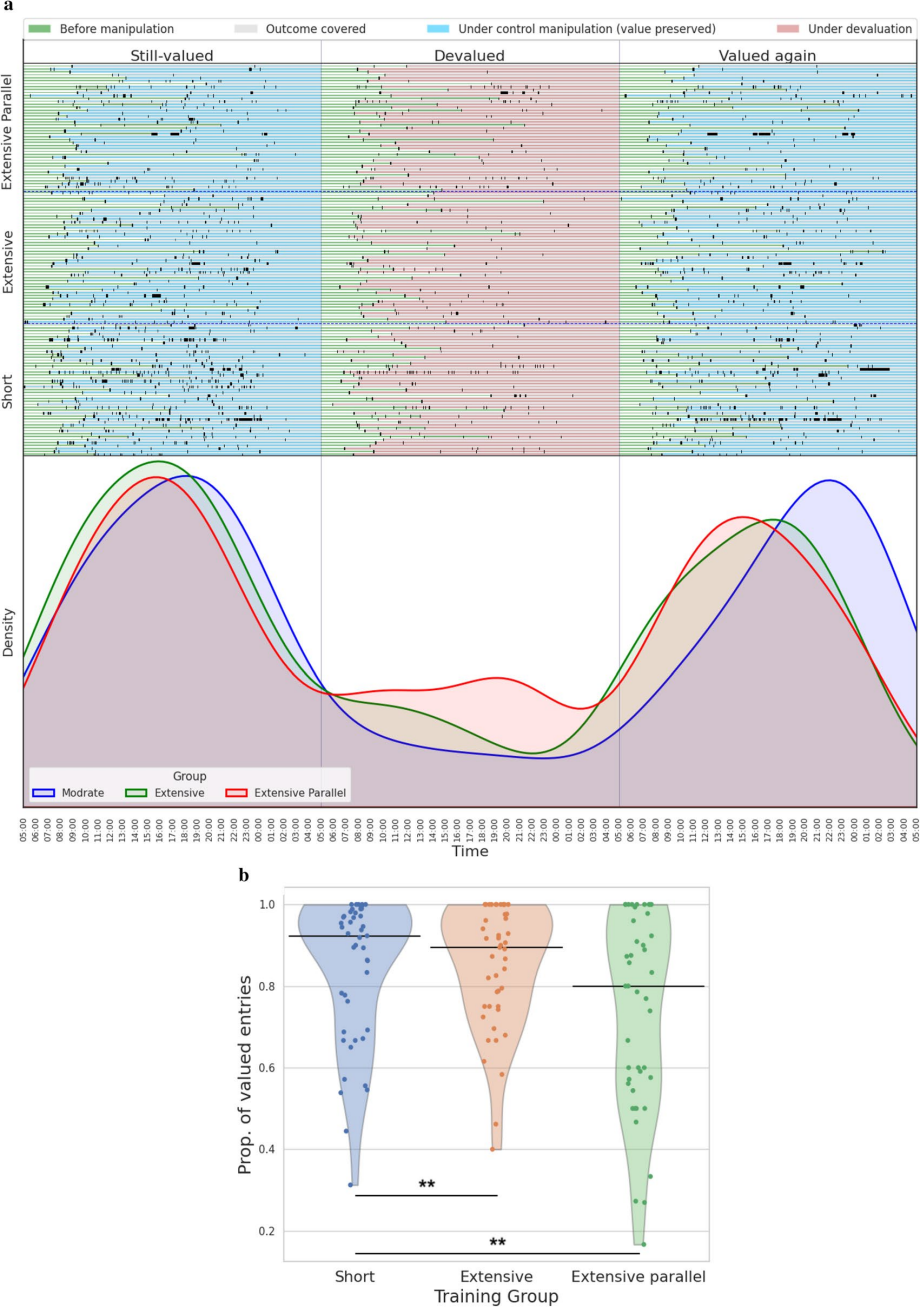
**Participants’ entries on the main manipulation days.** (**a**) On the upper part: a raster plot depicting participants’ entries (each vertical line represents an entry) throughout the three consecutive main manipulation days. Groups are separated by the dashed blue lines. On the lower part: the (relative) density of these entries across each group. (**b**) Relative proportion of valued (on the day before devaluation) vs. devalued entries ($\frac{still\ valued\ }{still\ valued+ devalued}$). The horizontal lines represent the median. Statistical significance indicated here was extracted from the relevant simple interaction effects of our main analysis, that is, the negative-binomial mixed-model (with a “quasi-Poisson” parameterization). * “Extensive parallel” refers to the extensive training group with additional parallel control manipulations (in the first week)

We found that participants were more likely to respond habitually following extensive training than after short training (Fig. 3), as indicated by a significant group × manipulation interaction (χ^2^_4_ = 12.85, *p* = 0.012) and significant simple interaction effects (Table 1). Simple interactions between pre-devaluation versus devaluation manipulation levels and the different groups indicated significantly less sensitivity to outcome devaluation (a hallmark of habitual behavior) in both the extensive training (*p* = 0.007) and the extensive training with parallel manipulations (*p* = 0.002) groups relative to the short training group (but not between the two extensive training groups; *p* = 0.529). This suggests that this procedure is able to capture the effects of training duration on habit formation/expression. We also found a simple group effect indicating that participants in the extensive training with parallel manipulations group entered less on the pre-devaluation day than participants in the short training group at *p* < 0.001. This a priori difference may have limited the extent to which these participants could manifest goal-directed behavior on one hand, but on the other hand made it conceptually easier for them to reduce their “habitual” entries (following devaluation), as less behavioral adaptation was required of them.

**Table 1:** Note: This data is mandatory. Please provide

	Entries		
*Predictors*	*Log-Mean*	*std. Error*	*p*
(Intercept)	3.76	0.15	<0.001 ***
Manipulation [Devaluation]	-2.14	0.18	<0.001 ***
Manipulation [Control - post]	-0.23	0.12	0.045 *
Group [Extensive Training]	-0.35	0.21	0.105
Group [Extensive Training - Parallel week1 manipulations]	-0.83	0.23	<0.001 ***
Manipulation [Devaluation] * Group [Extensive Training]	0.67	0.25	0.007 **
Manipulation [Control - post] * Group [Extensive Training]	0.05	0.18	0.778
Manipulation [Devaluation] * Group [Extensive Training - Parallel week1 manipulations]	0.85	0.27	0.002 **
Manipulation [Control - post] * Group [Extensive Training - Parallel week1 manipulations]	0.04	0.20	0.826

Mixed-model negative binomial regression analysis (with a “quasi-Poisson” parameterization of the variance) of participant entries following manipulations as explained by manipulation type, group, and their interactions. Control pre-devaluation manipulation and the short training group were used as reference levels

The *p*-values are the significance level

We also explored whether the proportion of participants with no entries following outcome devaluation (conceptualized as zero habitual responses) was larger following short versus extensive training. We found no significant effect between the short and either of the extensive training groups (χ^2^s_2_ < 2.20, *p*s > 0.138). Surprisingly, results were descriptively in the opposite direction. We presume this may have resulted from the requirement for a minimum of five daily entries we imposed on participants (see “Experimental procedure”). Participants’ confidence in and motivation to fulfill this requirement may have been different following a short training compared to extensive training, such that participants in the short training groups were willing to “pay” to make sure they stayed in the game.

### Manipulation checks

**Fig. 4:**
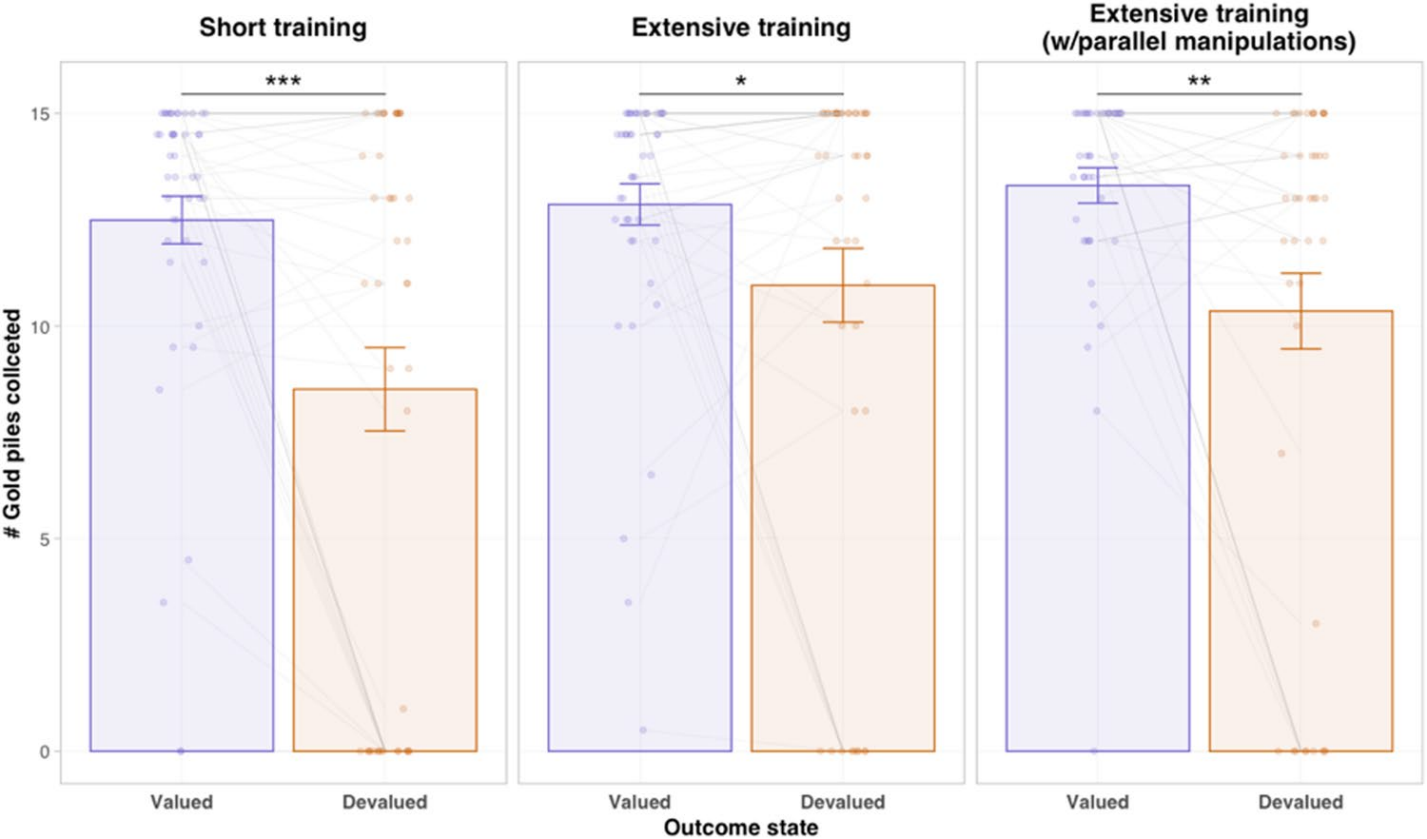
**Manipulation check of the outcome devaluation procedure.** Mean gold piles collected during a 5-second period of free gold collection following outcome devaluation and control (value unchanged) manipulations. Each press (i.e., touching the screen) cost 10 units of gold and each pile was worth 15 units. Error bars represent ±1 std error of the mean (SEM)

To verify the effectiveness of the outcome devaluation manipulation, we conducted two tests. First, we compared the amount of gold piles collected in the cave mini-task across all participants (Fig. 1b) following devaluation versus control manipulations (averaged across the days before and after outcome devaluation). As predicted, participants tended to collect significantly less gold following outcome devaluation (*t*_132_ = 5.66, *p* < 0.001; Fig. 4). A follow-up mixed-model analysis of variance (ANOVA) that included group, manipulation type, and their interactions as predictors confirmed that this change was similar across groups, as manifested in no group effect (*F*_2,130_ = 1.86, *p* = 0.16, ω^2^ = 0.01) or group × manipulation type interaction (*F*_2,130_ = 1.36, *p* = 0.261, ω^2^ = 0.002). This indicates that the duration (and type) of training did not lead to any discernible differences in participants' comprehension of the value manipulations. As expected, we found a significant manipulation type effect (*F*_1,130_ = 32.17, *p* < 0.001, ω^2^ = 0.08). For an additional test that verifies that the decrease in entries following devaluation can be attributed to sensitivity to outcome devaluation rather than a natural decline in engagement over time as the experiment progressed, please refer to the supplementary materials and Supplementary Fig. 2.

### Establishing an individual behavioral adaptation index

**Fig. 5:**
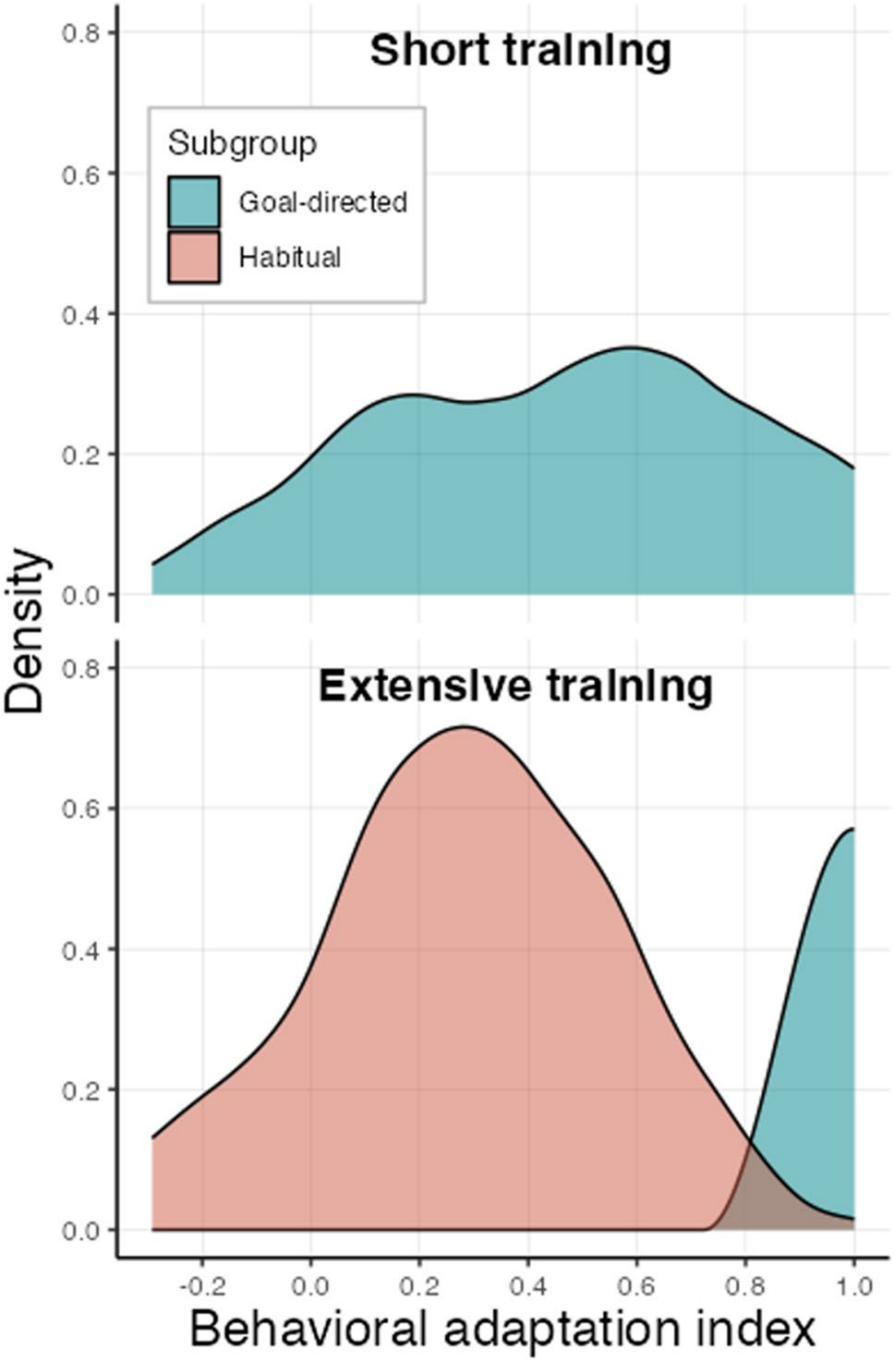
**Cluster analysis of the behavioral adaptation index.** Distribution of the identified clusters (latent subgroups) of the behavioral adaptation index. For each group (extensive training groups were combined), we fitted *k* = 1 or 2 clusters using a finite mixture-modeling analysis and chose the number of latent clusters that was most likely to generate the data (based on lowest BIC)

In order to test further hypotheses regarding factors involved in habit formation/expression, we formulated and preregistered an individual behavioral adaptation index (can also be referred to as a goal-directed behavior index; see “Data analysis” for more details about its calculation). This index ranges from −1 to 1, where 1 represents utter goal-directedness (no habitual entries), and values around 0 indicate habitual responding. We compared this index between the two extensive training groups, and since there was no significant difference (*t*_86_ = 1.50, *p* = 0.138 mean extensive training group = 0.51, mean extensive training with parallel manipulations group = 0.39), we collapsed their data for all subsequent analyses. We then tested whether the expected difference between the short and (combined) extensive training group is significant to evaluate the validity of our measure. This yielded no effect (*t*_131_ = 0.16, *p* = 0.872; mean short training group = 0.47, mean combined extensive training group = 0.45), suggesting this index might not be optimal to capture the full structure of the data. To further examine this index, we tested whether one or two latent subgroups would better explain its distribution within each group. To this end, we ran an exploratory finite mixture modelling analysis (Leisch, 2004). Based on the smaller Bayesian information criterion (BIC), we found that one cluster (of generally goal-directed participants) most likely produced the data in the short training group, whereas the data in the (combined) extensive training group was most likely formed by two latent clusters, consistent with the interpretation of one habitual and one goal-directed subgroup generating the data (Fig. 5).

### Unique smartphone app insights: Engagement patterns and habit formation

**Fig. 6:**
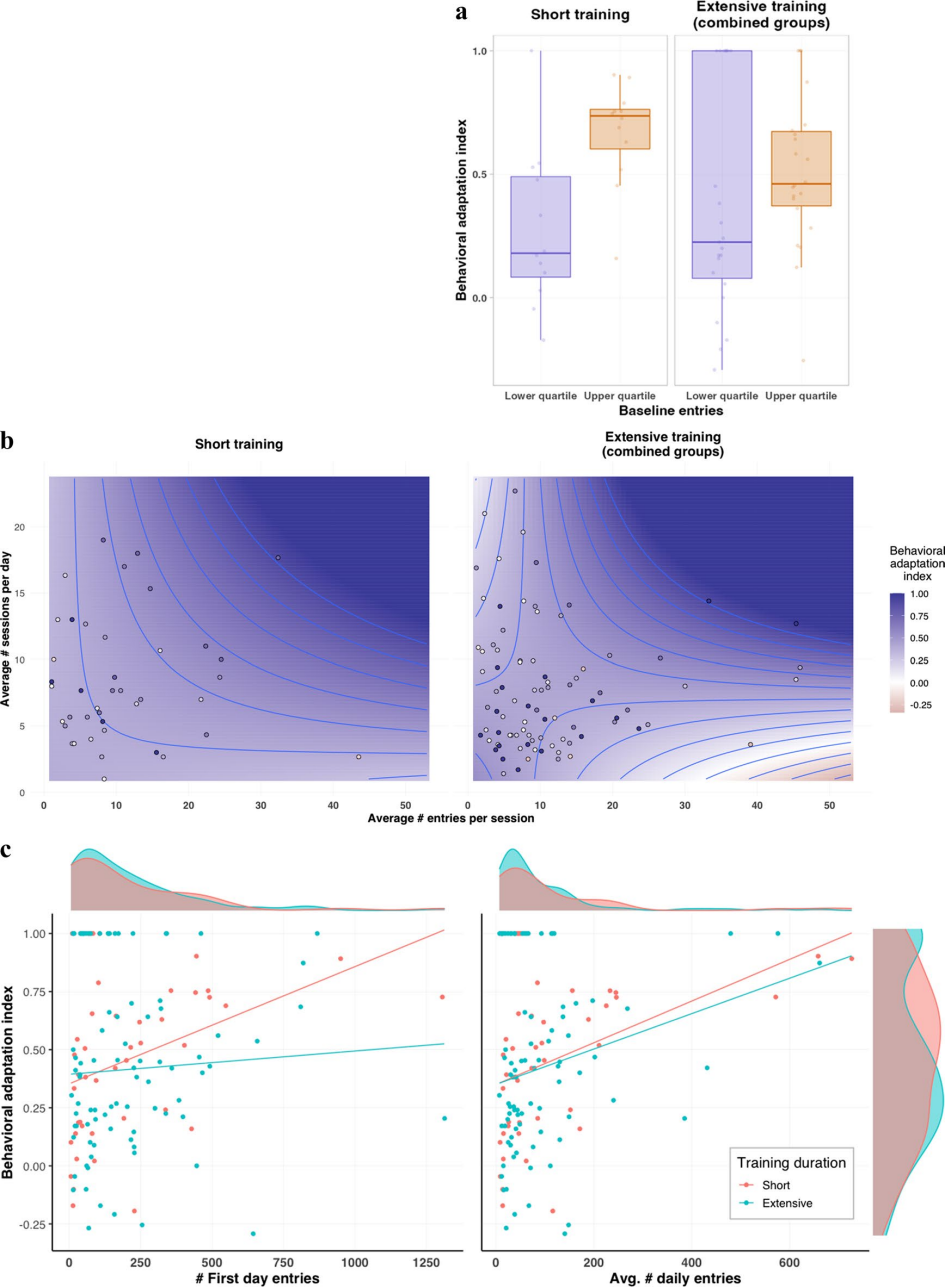
**Effects of engagement measures on habit expression.** The individual behavioral adaptation index, used as the dependent measure, can range between –1 and 1, where values around 0 represent habitual responding, and higher values represent goal-directed behavior. (**a**) Effects of high vs. low baseline rates (upper vs. lower quartile) as measured on the pre-devaluation day (following the control manipulation). (**b**) Effects of self-engaged spaced and massed training measures (based on inferred participants’ self-initiated session). The contours are portrayed according to the predicted values from a rank-based regression. Values were set to 1 if their prediction was larger. For visualization purposes, we set an upper bound of three standard deviations from the mean for both measures (resulting in four participants’ points not presented). (**c**) Effects of the number of entries on the first day and (left) and of the average daily entries (right). The regression lines are portrayed according to the predicted values from a rank-based regression (used for an exploratory analysis)

We conducted a series of analyses to examine the relationships between different aspects of engagement rates and patterns (represented by different indices) and habit expression. First, to test the relationship between baseline engagement rates and habit expression, we extracted the lower and upper quartiles of the data according to the number of entries committed by the participants on the pre-devaluation day (following the control manipulation). We then ran a rank-based regression on the behavioral adaptation index with group (short vs. [combined] extensive training), quartile (lower vs. upper), and their interaction as independent variables. We did not find a differential effect of baseline engagement rates on habit expression following short versus extensive training (specifically, we hypothesized that low baseline rates will promote more goal-directed behavior after short training vs. extensive training, and vice versa for high baseline rates), as indicated by the lack of an interaction effect (*F*_1,65_ = 2.26, *p* = 0.138). Instead, we found that baseline engagement rates modulated habit expression in both groups, such that high baseline engagement rates were associated with reduced habit expression (*F* = 13.40, *p* < 0.001, for the quartile main effect; quartile *B =* 0.45, *p* = 0.001; Fig. 6a).

The nature of our real-world free-operant task allows participants to not only determine their engagement rates but also their engagement patterns with the app. Participants tended to form self-initiated micro-sessions during which they committed multiple entries. We exploited this fact to estimate two measures we deem to represent the levels of spaced and massed training engaged by participants. To this end, we calculated the time interval between consecutive entries (inter-entry interval). Consecutive entries that occurred within an interval of less than 300 seconds were considered part of the same self-initiated session. We then calculated for each participant their average number of daily sessions and average number of entries per session. We entered these two measures along with group (short vs. [combined] extensive training) and all possible interactions as independent variables to a rank-based regression model on the behavioral adaptation index. We did not find an indication of differential effects on habit expression following short versus extensive training duration exerted by the self-initiated session indices (no three-way interaction and no relevant two-way interactions, *F*s_1,125_ < 1.80, *p*s > 0.182). Nevertheless, we found a main effect of the average number of entries per session (*F*_1,125_ = 5.19, *p* = 0.024), a trend of the average number of daily sessions (*F*_1,125_ = 3.87, *p* = 0.051), and importantly, an interaction between these two measures (*F*_1,125_ = 11.19, *p* = 0.001). The direction of these effects indicated that an increase in either of these indices, along with an additional mutual amplification, were associated with reduced habitual responding (i.e., with greater adaptation to outcome devaluation; averaged entries per session β = 0.18, *p* = 0.017; averaged daily sessions β = 0.16, *p* = 0.041; and their interaction β = 0.38, *p* < 0.001; Fig. 6b). This finding suggests that an increase in voluntary massed and spaced training, which probably reflects general engagement rates/motivation, synergistically contributes to one’s ability to deploy goal-directed behavior when such is preferred.

We also tested whether the number of entries performed on the first day of the task and the average of daily entries across the entire task (excluding the outcome devaluation day) predict habit formation. In our preregistration we planned a correlational (individual differences) analysis only across participants in the (combined) extensive training group. Correlating neither of these measures with the behavioral adaptation index resulted in significant effects (first day entries: Spearman *r*_86_ = 0.07, *p* = 0.247; average daily entries: Spearman *r*_86_ = 0.12, *p* = 0.126). We then conducted an exploratory analysis using the data from both groups. For each of these entry factors we ran a rank-based regression on the behavioral adaptation index in which we used the entries factor (either the first day entries or average daily entries), the group, and their interaction as independent variables. Running this analysis using the first day entries yielded a significant main effect of first day entries (*F*_1,129_ = 7.24, *p* = 0.008), but no group effect (*F*_1,129_ = 0.58, *p* = 0.446) and no interaction between group and first day entries (*F*_1,129_ = 2.93, *p* = 0.089). A similar analysis on the average daily entries showed a significant main effect of this measure (*F*_1,129_ = 14.33, *p* < 0.001) but no group or interaction effects (*F*s_1,129_ < 0.14, *p*s > 0.71). Performing more entries on the first day (β = 0.19, *p* = 0.007) and more averaged daily entries across all days (β = 0.30, *p* < 0.001) were associated with greater behavioral adaptation (i.e., goal-directed behavior; Fig. 6c).

### Model-free and model-based reinforcement learning strategies and habit formation

**Fig. 7:**
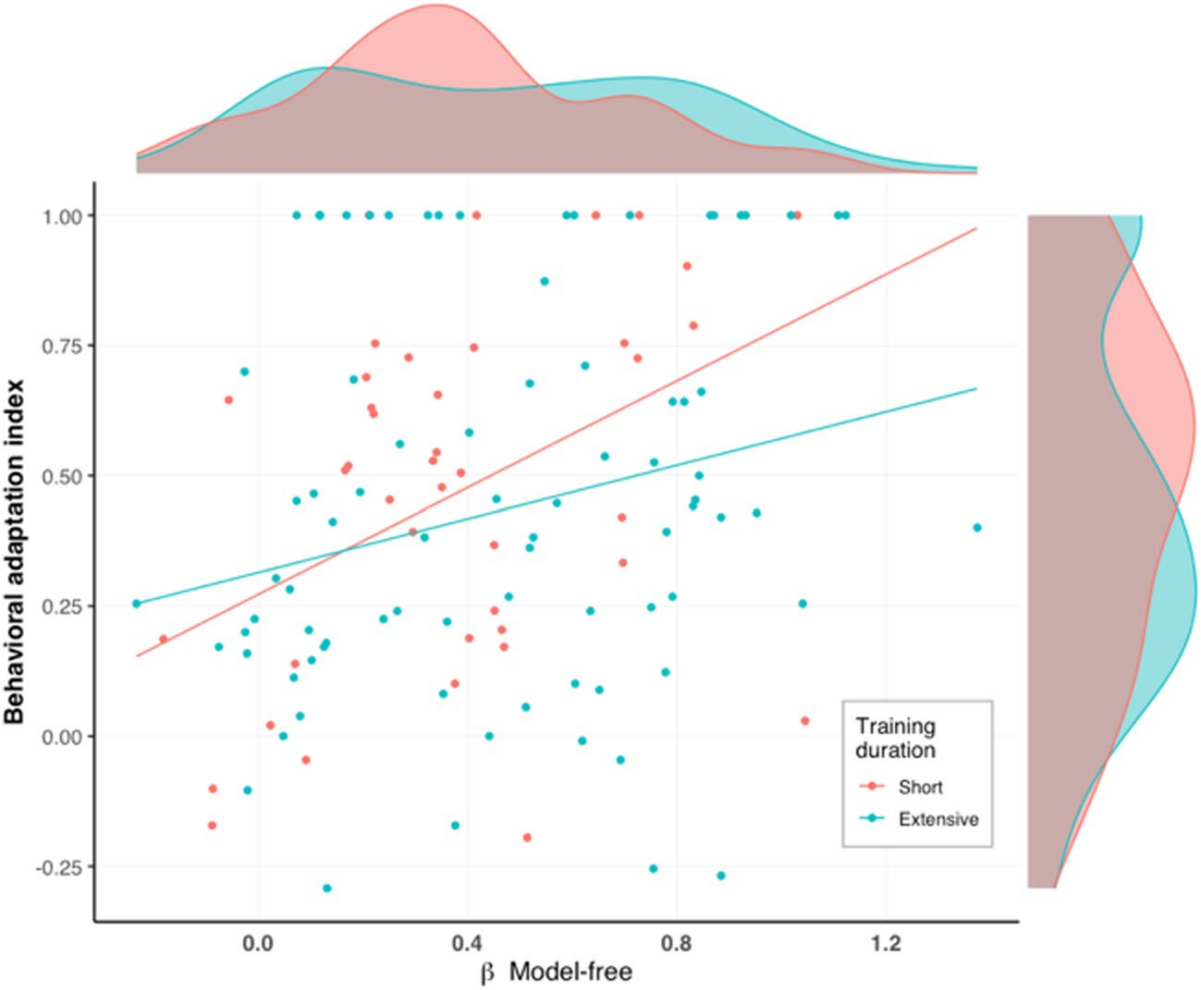
**Relationship between MF learning and habit expression.** Participants’ individual levels of MF learning (as extracted from the two-step task using a reinforcement learning computational model; Daw et al., 2011; Sharp et al., 2016) are illustrated along with their individual behavioral adaptation index as calculated from the app main task. The individual behavioral adaptation index ranges between –1 and 1, where values around 0 represent habitual responding, and higher values represent goal-directed behavior. The regression lines are portrayed according to the predicted values from a rank-based regression (used for an exploratory analysis). We consider this analysis of the relationship between these measures exploratory

MF and MB reinforcement learning strategies are commonly referred to in the literature as proxies of goal-directed and habitual action control, respectively. In an exploratory analysis, inspired by the work of Gillan et al. (Gillan, Otto, et al., 2015b), we examined how these indices, as extracted from the two-step task (Daw et al., 2011) (conducted by our participants following the completion of the mobile app part), were related to the behavioral adaptation index (and thereby to goal-directed and habitual action control) as measured in our task. We first fitted to the two-step task data a full reinforcement learning computational model designed to estimate the level of MB and MF strategies employed by each participant (see “Methods” and supplementary materials for details on the computational model). Subsequently, we used the MF and MB parameters (separately), along with group and their interaction, as predictors of the behavioral adaptation index in a rank-based regression. We hypothesized that MF action control is differentially associated with habit expression as a function of training duration, such that following a short training, MF learning is associated more with habits than following extensive training. In contrast, we found a main effect of MF action control (*F*_1,119_ = 8.66, *p* = 0.004) but no interaction or main effect of group (*F*s_1,119_ < 1.20, *p*s > 0.27). Interestingly, contrary to its traditional attributed correspondence with habitual behavior, we found that MF action control was positively associated with increased goal-directed behavior (β = 0.35, *p* = 0.001; Fig. 7). As opposed to MF, MB action control was not associated with sensitivity to outcome devaluation in our task (*F*s_1,119_ < 2.29, *p*s > 0.133 for the relevant main effect and interaction). Finally, the computational model also includes learning rate and perseverance parameters. We tested whether these parameters interacted with training duration and habit formation using a similar rank-based regression model. We found no such evidence for either of these parameters (*F*s_1,119_ < 1.37, *p*s > 0.244 for all relevant effects).

## Discussion

In this work, we established a novel paradigm for experimental habit induction in humans. This paradigm, implemented as a gamified mobile app, uses a new setting we named “real-world free-operant” that targets instrumental learning -related processes in a more ecological manner. We found that this procedure successfully demonstrated habit formation as a function of training duration, a long-standing challenge in the field of human action control, using the canonical index of reduced sensitivity to outcome devaluation. In addition, we found that individual engagement patterns played a substantial role in habit expression. Specifically, we found that more engaged individuals, despite being exposed to more training and more rewards, were more likely to remain goal-directed following outcome devaluation. Finally, in contrast to the common view in the field, we found in an exploratory analysis that MF learning was associated with goal-directed rather than habitual behavior, whereas MB learning was associated with neither.

Experimental habit induction in humans has constituted a significant hurdle for researchers in the field. Promising results of habit formation following extensive training were demonstrated in one study (Tricomi et al., 2009) (indexed by insensitivity to outcome devaluation), but a series of attempts were not successful in replicating those findings (de Wit et al., 2018; Gera et al., 2022; Pool et al., 2022). Other attempts targeting decreased sensitivity to outcome devaluation as a function of training duration were also unsuccessful (de Wit et al., 2018). This led researchers to pivot from the canonical outcome devaluation criterion and focus on alternative proxies of habitual behavior. Special emphasis was put on response time and related error rate effects. One study proposed that response time switch cost following devaluation (in choice trial settings) may indicate habitual behavior (Luque et al., 2020). Another study focused on error rates towards remapped associations as a function of training duration and given response time (Hardwick et al., 2019). These were important demonstrations of overtraining-induced effects, which may well approximate latent habit formation and capture key aspects of habitual action control (although see evidence for a different account of action slips under time pressure; Buabang et al., 2022). Still, establishing experimental habit induction using the canonical insensitivity to outcome devaluation criterion in free-operant settings is arguably more direct, more equivalent to the large body of animal research in the field, and more representative of how habits are formed and manifested in real life.

In the task we presented here, we introduced several features that may have contributed to the successful demonstration of habit formation as a function of training duration: (1) Addressing habit-related contextual (Bouton, 2021; Thrailkill & Bouton, 2015) and temporal aspects (Adams, 1982; Lally et al., 2010) is likely to have contributed to our observed effect. In addition to performing the experiment in their natural environment, participants used their own smartphones and were able to use the app whenever and to whatever extent they chose to for a relatively substantial period of spaced training, thus providing a similar experience to using everyday apps. (2) An important feature of our design was that, similar to successful demonstrations of habit formation in animals, and unlike previous attempts in humans, we used only one stimulus–response–outcome associative structure. The importance of this translational aspect is emphasized by findings showing that training animals on more than one instrumental associative structure has not led to habit formation (Colwill & Rescorla, 1985, 1986; Kosaki & Dickinson, 2010). It is possible that the presence of two similar associative structures in a similar general context encourages goal-directed control in the presence of choices between actions (discussed in Lingawi et al., 2015) or short trials (de Wit et al., 2018) and enhances habit expression in free-operant settings due to generalization of training on similar associative structures (de Wit et al., 2018; Gera et al., 2022; Pool et al., 2022). If this is indeed the case, avoiding the use of more than one associative structure may be key to successful experimental habit induction. (3) We trained participants on an action sequence rather than on a single action. Real-life instrumental behavior is rarely composed of a single action. If habits have evolved to automate adaptive behaviors and liberate cognitive resources, their formation is more beneficial, and thus perhaps more likely, when a sequence of actions is to be learned. Accordingly, it has been shown that action sequences may contribute to habit formation (Dezfouli et al., 2014). Some neuroscientific evidence also highlights the important role action sequences may play in habit formation. It has been shown that actions are chunked together at the neuronal level (in the striatum) as training progresses and habits are putatively formed (Graybiel, 1998). Additionally, the sensorimotor striatum, a region previously implicated in habit expression (de Wit et al., 2012; McNamee et al., 2015; Soares et al., 2012; Tricomi et al., 2009), increases its activity in tasks involving action sequences (e.g., Lehéricy et al., 2005; Poldrack et al., 2005), whereas in tasks involving a single action (in rodents), the number of neurons activated in this region was reduced over training (e.g., Barnes et al., 2005; Tang et al., 2007). While it is not feasible to quantify the unique or synergistic contributions of each of these task features in the present work, they could be targeted in future investigations using the task that we introduced here.

The unique session-free structure of our procedure, along with its 24/7 availability for a substantial period of time, allowed us to measure participants’ engagement (as a proxy of their motivation) in a unique manner, presumably more reliable and elaborate than is possible in typical laboratory or online session-based experiments. Even in traditional free-operant settings, participants are only “pseudo-free” to act in a manner that is aligned with their genuine motivation. They are “confined” to the experimental context and have only a very narrow range of potential actions they can perform during predefined limited timeframes. Our procedure was free of these limitations and thus captured participants’ engagement dynamics in a more comprehensive and complete manner. Here, examining the relationships between different engagement aspects and habit expression revealed novel and perhaps counterintuitive findings. Engagement patterns were shown to play a substantial role in habit formation/expression, such that more engagement was associated with greater goal-directedness. Higher engagement rates indicate a greater amount of (voluntary) training and more accumulated reward (with ratio reinforcement schedule), which, in contrast to our findings, may be more aligned with enhancing habit formation (e.g., Adams, 1982; Dickinson et al., 1995). We suggest that engagement patterns as manifested in our task capture an additional layer of motivational aspects that, when they are high, push in the opposite direction, perhaps by increasing the ability to resist habitual action control or by utilizing goal-directed action control when a response is no longer desired. Taken together, we suggest that engagement dynamics may encapsulate opponent processes that push action control in opposite directions. Verifying and characterizing these dynamics can be a target for future inquiry.

Another interesting finding was observed in our exploratory analysis of the relationships between MB and MF strategies and habit expression. MB and MF reinforcement learning strategies, typically measured with the two-step task (Daw et al., 2011), have been commonly regarded as proxies of goal-directed and habitual action control, respectively. Recent studies tested this equivalency by examining the relationships between MB and MF learning and habit expression as indexed by sensitivity to outcome devaluation. These studies found evidence for equivalence, at least to some extent, between the MB and goal-directed systems, but not between the MF system and either the goal-directed or habit system (Gillan, Otto, et al., 2015b; Sjoerds et al., 2016). We found that MF learning was associated with goal-directed behavior and MB learning was not associated with either system. This result is not aligned with either these recent investigations or the traditional view of MB and MF learning. It is difficult to determine what led to the difference in results between the aforementioned studies and the present work, since these previous procedures were different in multiple ways from our procedure. A possible explanation for the discrepancy between our findings and previous results is that in our procedure, high engagement rates (and hence, motivation) promoted both sensitivity to outcome devaluation and MF behavior. If this was indeed the case, it could imply that increased motivation primarily promotes the more simplistic MF strategy even if it does not maximize reward (although as we qualitatively observed, it may also increase MB learning).

### Limitations and potential improvements

A few limitations and potential improvements should be noted. One trade-off inherent to the real-world free-operant procedure is the uncontrollable and untraceable exposure to the stimulus (the app icon). A solution to tracking stimulus exposure may be feasible using tools that allow smartphone camera-based eye tracking (Valliappan et al., 2020). However, controlling for stimulus exposure while maintaining the real-world free-operant structure would be challenging. We presume that stimulus exposure, among other factors related to behavioral, psychological, and contextual individual differences which might be strongly manifested in such a procedure, may add a substantial amount of generally unwanted variance. This joins our general proposition that the expression of habits, especially when they are no longer adaptive, is a subtle effect, and its stability is subject to a variety of factors. The observation that a subset of participants in the extensive training groups manifested sensitivity to outcome devaluation (which is in line with previous evidence; de Wit et al., 2018; Gera et al., 2022; Pool et al., 2022) supports this notion and perhaps indicates that habit formation is not a unitary deterministic process. We therefore propose that acquiring large datasets is of great value in further establishing the effects found here and exploring other factors implicated in habit learning.

A future improvement that may increase the utility of our app is establishing a better behavioral adaptation index that more comprehensively captures individual levels of goal-directed/habitual responding. The measure suggested (and preregistered) as an adaptation index seems to capture this only to some extent, and a better index may help gain more accurate findings about how factors of interest interact with action control.

Another target for future improvement is strengthening the robustness of the main manipulation check, that is, the cave mini-task employed following value manipulations. While participants reduced the amount of gold they collected following outcome devaluation compared to the control manipulations (Fig 4), attaining a stronger adaptation (greater reduction) is preferable. We learned from post-experiment debriefing that the lower-than-desired effect is at least in part because (1) some participants believed that this part was unrelated to the warehouse capacity (although explicitly instructed that it was), and (2) a few individuals opted to engage in the mini-task (collecting gold) due to its enjoyable and challenging nature, willing to endure the associated costs. Thus, to enhance the effectiveness of the manipulation check, it could be beneficial to place additional emphasis on the relationship between the gold in the cave and the condition of the warehouse, and to increase the negative consequences associated with that gold collection following outcome devaluation. Another approach would be to replace this mini-task with a simple question that verifies participants’ understanding of the task (see more details below in “New features and customizability: Expanding the app for future utilization”).

Finally, our procedure is limited in the extent to which it can be used to study the neural underpinnings of habit formation and expression. Since it is not applicable for real-time laboratory-restricted research tools like task-based functional magnetic resonance imaging (task-fMRI), it is not well-suited to capture the neural dynamics occurring while participants performing the task and action control presumably shifts from the goal-directed to the habit system. Nevertheless, in combination with tools like resting-state fMRI and diffusion tensor imaging (DTI), we suggest that our procedure has the potential to enhance our understanding of the neural mechanisms involved in habits. This could be achieved by tracking functional and microstructural neural plasticity induced by our task, as well as through the examination of functional and structural neural correlates of individual habit propensity.

### New features and customizability: Expanding the app for future utilization

A primary objective of this study was to introduce a novel methodological framework we named the real-world free-operant task, alongside a tool designed to facilitate the implementation of experiments utilizing this framework. To make it accessible and relevant to a wide range of the researchers in the multiple branches of behavioral, psychological, and neural sciences, we constructed a highly modular version of the app, allowing researchers to easily integrate a variety of features and tune a wide range of parameters. Furthermore, to ease the process of modifying the app, we created an online tool that enables one to conveniently set the task features and parameters (https://ranigera.github.io/RWFO_app_setup). Below we describe a non-exhaustive list of the modifiable features and tunable parameters we implemented, and, where relevant, we mention the motivation to include them and/or their potential pros and cons compared to the ones used in this study:

(i) It is possible to adjust the number and timing of cloud appearances (outcome masking) and warehouse manipulations (outcome devaluation and control). This allows one to introduce these features in a more regular and/or flexible manner to participants. Doing so can help reduce the saliency of outcome devaluation and thereby mitigate potential enhancement of goal-directed behavior. This flexibility will be particularly beneficial for studies employing similar training durations for all participants, where more frequent and flexible exposure to these task features would not lead to an unbalanced design between different experimental groups.
(ii) The manipulation check we used (the mini-task of gold collection in a cave) has several advantages. It is relatively fun and challenging and could increase general engagement. In addition, it generates a behavioral parametric measure which could be useful for certain use cases. Nevertheless, for researchers that might opt for a more simplistic and explicit test, we added an alternative configuration by which, following value manipulations, participants are simply prompted to type the current status of the warehouse.
(iii) In retrospect, the warehouse capacity was framed suboptimally. The warehouse is supposedly completely emptied at the beginning of each day, but then on some days it becomes half full after five entries but never gets completely full no matter how many more entries have been committed afterwards. On another day, it becomes completely full after five entries. Crucially, it appears that participants' performance in our study remained unaffected. This is probably due to a slightly different wording used in the instructions and comprehension test with respect to the warehouse status. In these parts, participants were instructed that sometimes they would receive a report that the warehouse had become “partially” (rather than “half”) full and that as long as it was not completely full, they could continue to accumulate gold. This seems to have helped us to successfully convey the intended meaning of devalued versus still-valued (control) phases. Nonetheless, in the new app version, to increase the framing quality and prevent potential confusion, we changed the instructions and the daily message on emptying the warehouse to convey the message that the cargo spaceship is sometimes able to empty just some of the gold in the warehouse and not all of it. This should make the warehouse status messages (or not getting messages) more believable and consistent.
(iv) Explicitly reminding participants of the value status of the outcome or the counterproductivity of the (habitual) responses committed following outcome devaluation could prompt the goal-directed system to gain control and thereby reduce sensitivity to capture habit formation. Nevertheless, indicating that a potential outcome is no longer valuable following responses towards that outcome is a worthwhile feature that might be preferable in some cases and that could increase the confidence in interpreting post-devaluation actions as habitual. We thus added an option to include a message shown following post-devaluation sequence completions that says, “A reminder: the warehouse is full and cannot store more gold today.”
(v) The fixed reward pattern on the first two daily entries (first non-rewarded, second rewarded) may not be necessary. We thus added the option to remove any such pattern or to replace it with a more flexible pattern of two random rewarding entries out of the first five entries.
(vi) To enable researchers to use a wide range of reinforcement patterns and investigate their effects on behavior and learning, we have incorporated two fundamental functionalities. These include the option to set a random interval reinforcement schedule and the ability to incorporate aversive outcomes (i.e., losing gold).
(vii) Using multiple learning contexts with our framework could be a desired feature for numerous use cases. Since the app is implemented as a progressive web application (PWA), it allows for easy installation of multiple instances on the same device. Each instance can have slight variations in background/stimuli images and/or task parameters, allowing for the creation of distinct learning environments or contexts.

### Conclusions

In conclusion, we introduced here a novel ecological procedure, implemented as a gamified smartphone app, for experimental habit induction. This app is a hybrid between field and laboratory experimental settings, seeking to draw the best from both worlds. Our unique paradigm introduced a new real-world free-operant framework by which participants can freely engage whenever and as much as they liked. Using this paradigm, we successfully demonstrated the sought-after effect of habit formation as a function of training duration. The real-world free-operant structure of the app also allowed us to track engagement dynamics from several different angles and demonstrate its positive relationship with maintained sensitivity to outcome devaluation. Finally, in an exploratory analysis we found contradictory effects to the common view conceptually paralleling MB learning to goal-directed behavior and MF learning to habits. Our app can be used in the future, ideally by collecting large datasets, to enrich our understanding of the psychological and neurobehavioral mechanisms underlying habit formation in humans “in the wild.” Furthermore, it is well suited for studying a plethora of other instrumental behavior -related questions in a naturalistic manner.

## Materials and methods

### Participants

We collected data from 145 participants, randomly assigned to three experimental groups. Ten were excluded due to not fulfilling a requirement of at least five daily entries across all the days of the experiment. One was excluded due to a technical error (one of the manipulations along with its subsequent manipulation check appeared twice in a row on two consecutive entries). One participant was excluded due to a pattern of what appeared to be nonhuman, automated entries (see details in the supplementary materials). Thus, our final sample of valid participants included 133 participants (91 female) aged 18–38 (mean = 25.3, *SD* = 3.59), 45 in a short training group, 45 in an extensive training group, and 43 in an extensive training with parallel manipulations group (see “Procedure” for details about the groups).

Note that the 10 participants who did not reach the minimal daily entry requirement belonged to the extensive training groups (four and six in the extensive and extensive with parallel manipulations groups, respectively). This raises the question whether a survivor bias may have influenced our results. Two of these 10 participants did not commit their minimal entry requirement in the first few days and therefore would have been excluded regardless of their group assignment. This leaves eight out of 100 participants who began their participation in these groups (8%) who may have theoretically contributed to a survivor bias. This is a relatively low rate, especially for such a longitudinal real-world study, and we presume it had little, if any, effect on our results.

We administered the study in two batches, a small one (yielded 34 valid participants out of 40) followed by a large one (yielded 99 valid participants out of 105). Our sample size is larger than our planned (and preregistered) target sample size of 90 valid participants (30 per group). This stems from the fact that we ran the study in batches, and based on previous pilots, we overestimated the number of participants that would not start their participation and in particular the number of excluded participants (which was considerably less than we had anticipated). Note that this target sample size was rationalized as a compromise between our desire to acquire as large a dataset as possible, to be able to detect even subtle effects in a relatively noisy environment, and the resources available for this well-paid study.

As part of the recruitment process, we prescreened participants to ensure they have constant access to their smartphone (including on weekends) and to the internet and that they are not planning to travel abroad or replace their smartphone within a period of one month from the day scheduled for the beginning of their participation.

For the two-step task, performed following the main part of the experiment (see Post-experimental procedures), out of the 133 valid participants, we were able to collect data from 127 participants. The data for six participants were not collected due to technical problems (either they could not open the task on their personal computer or they performed the task but the data were not successfully saved to our server). Another four participants were excluded from analyses involving data from the two-step task for failing to meet one or more predetermined behavioral exclusion criteria used to verify participants’ engagement in this task (see “Data analysis”).

### Experimental procedure

#### About the app

We devised and developed a progressive web application (PWA), named “Space gold”, compatible with both Apple iOS and Android-based smartphone devices, to be used as an experimental platform to ecologically induce and study habit formation and expression. We programmed the app in vanilla JavaScript, and for the instructions part we also used jsPsych (de Leeuw, 2015). We put an emphasis on gamifying the app to enhance the real-world naturalistic app-using experience and correspondingly attenuate the experience of participating in a scientific behavioral experiment. The app was designed to work both online and offline to maximize its availability to participants. Finally, to prevent parallel installation of the app on multiple devices, we implemented a mechanism that allowed a one-time-only installation through a designated link.

#### Installation and instructions stage

Participation in the experiment started on Sundays (the first day of the work week in Israel). This was done in order to retain uniformity across participants and avoid additional between-participant variance or confounding effects exerted by the different days of the week. Importantly, starting on Sundays ensured that none of the manipulation days (as we scheduled for the different experimental groups; Fig. 1c) would fall on the weekend, which we rationalized to generally constitute a substantially different temporal and most likely physical context.

Participants received an email with a personal installation link at 8:00 am on their scheduled starting date and were required to complete the installation and instructions part no later than 11:00 am. In practice, we allowed an extra hour (until 12:00 pm) to participants who were not able to complete it in time. The participants were required to install the app and place it in a specified position (on the second row from the bottom, as the second app from the right or on the center, in a four- or five- app–column interface, respectively; see example in Fig. 1a) on their main home screen (defined as the one they use the most). To ensure it was positioned as instructed, we asked the participants to send us a screenshot of their home screen following the installation. The participants were then required to enter the app and go over the instructions of the task (framed as a game), followed by an active demo, and then by a comprehensive comprehension test. The test was composed of 11 multiple-choice questions. Failing to correctly answer one or more questions required participants to repeat the test (after given the opportunity to go over the instructions and demo again). This cycle repeated itself until all questions were answered correctly. At 12:30 pm, after all the participants had completed the instructions part and became “active players,” we emailed them a copy of the instructions.

#### Real-world free-operant task

The general narrative of the game, as presented in the instructions, was that the player (participant) had the ability to send a spaceship to a distant planet rich with gold, called “the gold planet”, in order to search for gold. The gold they found was stored in a warehouse situated on the gold planet. Every 24 hours, all the gold acquired in the warehouse was taken to earth by a cargo spaceship, and the warehouse became empty and ready again to store gold. This happened automatically, and participants received a message stating that the warehouse had been emptied every day on their first daily entry (defined as the first entry following 5:00 am). Participants were instructed that the gold transferred to earth will be converted to real money they would receive at the end of the game. They were also informed that the warehouse could get full, and once that happened, they could no longer store gold in the warehouse until the next day, effectively rendering the gold worthless for the rest of the day (this was the outcome devaluation manipulation). To further increase participants’ motivation to acquire gold, they were told that every few days we would conduct a lottery for additional money prizes between participants who finished their participation and that their chances to win were directly proportional to the amount of gold they managed to acquire throughout the task. Note that some of the features in this task, particularly the reward and manipulations on its value, were inspired by a previous task (Gillsn, Otto, et al.,2015b).

Participants were assigned to three experimental groups varied in their training duration, i.e., the number of training days they had before the outcome devaluation manipulation, and in the number and schedule of their control manipulations (see details below in *Experimental groups*). To avoid any self-selection bias or different expectations regarding the task, all participants, regardless of their group assignment, were told that the experiment duration is not predetermined and that it may last between a few days and up to a month. Consistently, participants in all groups received exactly the same instructions.

##### Task structure

To send their spaceship to the gold planet, participants had to enter the app by pressing its icon on their smartphone (Fig. 1a). The app icon was effectively the stimulus/cue associated with an action and an outcome. After pressing the app, right as it opened, participants were presented with a quick animation of their spaceship landing on the gold planet along with an indication of a one-gold-unit cost they had to pay for each space travel to the gold planet (namely, for each entry). The cost was added to prevent participants from intentionally entering the app “just for fun” in periods when the outcome had lost its value (i.e., when the warehouse was full) and thereby to allow us to interpret these entries as habitual (outcome-insensitive) responses.

Following their entry to the app, participants had to remove a layer of ice from the ground before they could look for gold. To this end they were required to press on the lower half of the screen followed by a press on the upper half of the screen. Thus, together with the initial press on the app icon (to enter the app), participants had to perform a three-press sequence. This was mainly done to represent real-life habitual responding, which is typically composed of a sequence of actions rather than a single one, and to allow us to track partial responses (but since partial responses were highly uncommon, we did not analyze them). After completing the sequence pressing, a short animation of a bulldozer digging in the ground was presented, followed by a presentation of the outcome, and shortly after, the message “see you next time” appeared. The outcome was either 15 units of gold or a worthless piece of rock. We used a variable-ratio reinforcement schedule, where on each entry the chance of finding gold was 1/3 (VR-3). To retain participants’ engagement, in the case of not finding gold for six consecutive entries, the seventh entry was guaranteed to yield gold. In addition, we fixed the first daily entry on each day to result in a rock and the second to result in finding gold. We rationalized that this pattern would motivate participants to enter at least twice on their first daily interaction with the app. A typical trial/entry lasted around 8–10 seconds. Notably, participants were completely free to determine their pattern of engagement with the app, that is, they were free to enter the app whenever and as much as they liked 24/7 throughout the entire duration of the experiment. The only requirement we imposed in order to stay in the game was to enter at least five times a day. To constantly remind participants of this requirement, they received two reminder emails on each day of the experiment (at 8:00 am and 6:00 pm).

##### Outcome devaluation and control manipulations

On the third or tenth day of the experiment (depending on the experimental group; see details below), we introduced the outcome devaluation manipulation (Fig. 1b). On their fifth daily entry, participants were presented with an image of the warehouse completely filled with gold boxes, along with a message stating that the warehouse is full and that it could not store any more gold until it is emptied again (by the cargo spaceship). This meant that the outcome was devalued until the next day. A day before and a day after the outcome devaluation manipulation, we employed a control manipulation where a message (shown on the fifth daily entry) stating that the warehouse is half full was presented, along with an image of a partially full warehouse. This meant that gold could still be accumulated and thus retained its value. To ensure that participants paid attention to the manipulation message, they were required to enter a (random) three-letter code to proceed. In the event that participants exited the application without confirming the message, it reappeared upon their next entry, and so on, until confirmed by the participant.

The number of entries following the introduction of the outcome devaluation and control manipulations was used as our main dependent variable. To prevent the potentially confounding effects of the outcome presentation itself on the balance between goal-directed and habitual action control, we concealed the outcome. This occurred on each manipulation day, starting from the third daily entry and continuing until the next day. Concealing the outcome from the third entry was aimed to prevent, at least to some extent, a direct association between concealing the outcome and the outcome devaluation, as well as to habituate participants to concealed-outcome entries before any manipulation was induced. To implement this feature, as part of the task instructions, participants were informed that sometimes, due to bad weather conditions on the gold planet, they would not be able to see the outcome of their search (implemented as clouds covering relevant parts of the screen; see Fig. 1b). We emphasized that, other than not being able to view the outcome, everything else remained the same, including their chances of finding gold on each entry.

##### Manipulation check

At the instructions phase, participants were informed that sometimes when they entered the app to find gold, they might encounter a cave rich with gold, and that when that happened, they had 5 seconds during which they were able to collect gold piles (worth 15 units each, similar to the regular dug gold piles) by simply pressing on gold piles scattered across the screen. They were also instructed that each attempt to collect the gold inside the cave (i.e., each press) exerted a large 10-gold-unit cost. Inside the cave, there were 15 randomly positioned gold piles and 15 pieces of worthless rock. Participants encountered such caves immediately following each manipulation introduction (outcome devaluation and control manipulations), that is, immediately after they had confirmed the message about the state of the warehouse. We embedded this mini-task as a manipulation check for the outcome-devaluation procedure. The cave was used as a different setting than the regular training, and we measured the number of gold piles participants collected in them as an indication of their awareness of the current outcome value.

##### Experimental groups

We varied the duration of the experimental task (and thereby of the training) across the three experimental groups (Fig. 1c). The task lasted 4 days in the short training group and 11 days in each of two extensive training groups. We initiated the experiment for the short training group exactly a week following the initiation of the experiment for the extensive training groups. The 7-day interval was determined so that participants in all groups undergo the manipulations on the same particular days.

In one of the two extensive training groups, we added control manipulations on days parallel to the three manipulations in the short training group (i.e., days 2–4). This was done to account for the option that the mere encounter with “non-regular” trials only after a relatively long period (i.e., only in the second week) would exert its own confounding effects. This also enabled us to obtain direct comparable manipulation days with the short training group manipulations, but without outcome devaluation.

#### Post-experimental procedures

After completing the real-world free-operant task, we asked participants to perform an additional part on their personal computers. First, they underwent the two-step sequential decision-making task (Daw et al., 2011) to estimate their individual tendency to utilize MF and MB reinforcement learning strategies. These learning strategies were previously commonly referred to as proxies of habitual and goal-directed action control, respectively. We adapted an online version of this task from the Experiment Factory Battery (Sochat et al., 2016). Briefly, in this task, participants could choose between two actions in a first state. Each first state action commonly (70% of the times) led to one of two possible second states and rarely (30% of the times) to the other in a respective manner (i.e., the common–rare probabilities are reversed between the two first state actions). In either of the second states, participants were required again to choose one of two actions (different pairs in the different second states) which could have led to winning a reward. Reward probabilities of the second states’ actions were gradually changed throughout the task. After completing this task, participants were asked to fill out a battery of psychological questionnaires. The link for this part (two-step task and questionnaires) was sent to the participants after they had completed the real-world free-operant task on the following Friday at 8 am. We asked participants to complete it as soon as possible and no later than the following Monday.

## Data analysis

All statistical analyses were carried out using R programming language (R Core Team, R Foundation for Statistical Computing, Vienna, Austria, 2021) and Python programming language (Van Rossum et al., 2009). The latter was also used for data extraction and parsing.

### Main analysis

To test the effect of training duration on habit formation (as estimated by sensitivity to outcome devaluation) we ran a full mixed-model Poisson regression, implemented using the glmer function from the lme4 package in R (Bates et al., 2015). In the model, we entered the number of entries following manipulations as the dependent variable. As independent variables, we entered the group and the manipulation (that is, the outcome devaluation and the control manipulations induced the day before and the day after that) factors and their interaction to the model. Participant was entered as a random effect. Accordingly, the regression was formulated as follows (in lme4 syntax):

$$\textrm{entries}\sim \textrm{manipulation}\ast \textrm{group}+\left(1|\textrm{participant}\right)$$

Note that unlike the model conceptualized in the preregistration, this model does not include a random slope since there is only one value per within-participant condition, a case for which a random intercept model should be used. Since running the Poisson regression resulted in a large overdispersion (tested using the model’s sum of squared Pearson residuals, as implemented by the performance R package; Bolker, 2017; Lüdecke et al., 2021), we tested which of three models aimed to handle over-dispersed count data ( Bolker, 2022) would best fit our data. The three candidate models, implemented using the glmmTMB R package (Brooks et al., n.d.), were an observation-level random effects model, a negative binomial mixed-model regression with a quadric parameterization of the variance (NB2), and a similar model with a linear (“quasi-Poisson”) parameterization (NB1). For model comparison, we used LOOCV and chose the model with the lowest mean squared error (MSE).

We also tested the hypothesis that the proportion of participants without a single habitual entry (“utterly goal-directed participants”) would be larger following a short training versus extensive training by comparing these proportions between the short and each of the extensive training groups using a two-proportion *z*-test (with Yates continuity correction).

### Calculating and validating a behavioral adaptation index

To test the involvement of other factors in habit formation/expression, we calculated an individual behavioral adaptation index, which measures the relative change in responding following outcome devaluation for each participant. As preregistered, the index was formulated as follows:

$$\frac{\sqrt{\mu valued}-\sqrt{devalued}}{\sqrt{\mu valued}+\sqrt{devalued}}$$

This individual behavioral adaptation index measure was calculated by first averaging the number of entries on the (control) pre- and post-outcome devaluation days (i.e., when the outcome retained its value) in an attempt to capture a potential general reduction in engagement rates. We then transformed the data by calculating the square root of the number of entries on the (averaged) valued days and the devalued day to assign less weight to highly inflated numbers (and larger relative weight to small numbers). This index ranges from −1 to 1, where 1 represents no habitual entries (i.e., “absolute” sensitivity to outcome devaluation), and values around 0 indicate habitual responding (i.e., “absolute” insensitivity to outcome devaluation). Relatively large negative values, which are conceptually irrational, were not present in our data.

We used a *t*-test to compare the calculated behavioral adaptation index (see “Results") between the two extensive training groups. As there was no significant difference between the two groups, we collapsed their data together for all subsequent analyses relying on this index. We refer to this merged group as the combined extensive training group. We used a *t*-test to compare the behavioral adaptation index between the short and the combined extensive training groups. Note that (as stated in our preregistration) as opposed to the regression analysis on the raw entry data, this analysis was not a part of the main confirmatory analysis of the emergence of habits as a function of training duration. Rather, this analysis was aimed to examine how the behavioral adaptation index, that we formulated in order to create a single value indicator of individual habit expression, is different between groups with different training duration. To further test this index and characterize the data by identifying latent subgroups within each training group, we ran a finite mixture modelling analysis using the Flexmix R package (Leisch, 2004) on this measure. We ran the model using *k* = 1 or 2 clusters and considered the number of clusters (i.e., subgroups) that yielded the lowest Bayesian information criterion (BIC) as the number of underlying latent subgroups that most likely gave rise to the data in each group.

### Engagement and habit formation

To test whether baseline engagement rates were differentially associated with habit expression following short versus extensive training, we extracted the lower and upper quartiles of the data according to the number of entries following the control manipulation (i.e., warehouse was half full) on the day before outcome devaluation. Note that the extraction was done separately within each group, but we verified that the results maintained the same pattern when the extraction was performed across all participants together. We ran a rank-based regression using the Rfit package (Kloke, 2012) on the behavioral adaptation index as the dependent variable, the group and quartile (upper vs. lower) factors, and their interaction, as independent variables.

We also used a rank-based regression model to test the effects of the average daily amount and density (with respect to the number of entries) of participants’ self-initiated sessions (see Results for details on how these measures were inferred). These two measures along with group and all possible interactions were entered as independent variables and the behavioral adaptation index as the dependent measure. Note that to have more than only one “regular” training day (namely, with no manipulations) in the short training group, we included the control manipulation days in the calculation of the session indices. We made sure this had not affected our findings by running the same analysis on self-initiated session indices calculated after omitting all manipulation days (see supplementary materials).

Finally, we examined whether individual differences in the number of entries to the app on the first day and the average number of entries across all task days (except for the outcome devaluation day) were associated with habit expression (tested separately for each of the entry measure). For this purpose, we used a one-sided Spearman correlation test between each entry measure and the behavioral adaptation index. As stated in our preregistration, this was done only for participants in the extensive training group, mainly as the average number of daily entries measure relies on more data. Nevertheless, we accompanied this analysis with an exploratory analysis. For this analysis, we used the entire dataset and ran a rank-based regression on the behavioral adaptation index for each of the entry measures as a factor along with group and their interaction.

Note that for each rank-based regression involving continuous independent variables, we first normalized the data and coded the short training group as −1 and the (combined) extensive training group as 1 (effect coding). Another thing to note is that the data used for quantifying some of the engagement aspects we targeted (e.g., the baseline engagement rates from which we extracted the lower and upper quartiles) was also used in part for the behavioral adaptation index calculation. Thus, while conceptually (apart from the case of zero entries) the behavioral adaptation index was free to go in either direction regardless of the engagement measure value, they should not be viewed as completely free of inherent structural dependency.

### Manipulation checks

To test that participants indeed understood the manipulations, we conducted two tests. First, we calculated the average number of gold pieces collected in the cave following the control manipulations in the days before and after outcome devaluation across all participants and compared it with the gold collected following outcome devaluation using a paired-sample *t*-test. To confirm that such effects were similar across groups, we also ran a mixed-model repeated-measures ANOVA on the mean number of gold piles collected with group as a between-participant factor and the preceding manipulation type (outcome devaluation or control) as a within-participant factor. Second, we performed a paired-samples *t*-test across all participants to compare their engagement with the app on the outcome devaluation day and on the subsequent day to verify that in general it went up again once the outcome had regained its value. The measure used for this test was the square root of the relative proportion of entries committed following each manipulation day (that is, the day of outcome devaluation and the adjacent before and after days). This measure normalizes the entries within each participant in an equivalent way to our main behavioral adaptation index (if we would have used the same further calculation of the relative difference; see above). We also repeated this test separately within each group. The results of this analysis are described in the supplementary materials.

### MF and MB reinforcement learning strategies and habit formation

To verify participants’ engagement in the two-step task, we applied the exclusion criteria used by Gillan et al. (Gillan, Otto, et al., 2015b), who also used a variant of the two-step task online. Specifically, we excluded two participants who missed more than 10% of the trials and two participants due to implausibly fast response time (−2 SD from the group mean). Note that we also set a very slow reaction time (+2 SD) and pressing on the same key > 90% of the times as exclusion criteria, but these were not met by any participant. To test the relationship between MB and MF action control as inferred from the two-step, along with other parameters of interest (learning rate and perseverance), we fitted a full reinforcement learning computational model based on transitions, rewards, and participants’ choices in the two-step task. We formulated our computational model as a variation of the model used by Daw et al. (2011), by which the level of MB and MF strategies employed by each participant are estimated, with similar adaptations as those implemented by Sharp et al. (2016) with respect to the within-participant-level part of the model (see a full description of the model in the supplementary materials). We estimated and extracted the following parameters for each participant: reliance on MB and MF learning strategies at the first stage, reliance on MF learning strategy on the second stage, perseverance, and learning rate. The computational model parameters were estimated through the No-U-Turn sampler-based Hamiltonian Monte Carlo as implemented in the Stan (Stan Development Team, 2021) Bayesian inference engine. We then entered each of the extracted parameters as an independent variable along with group and their interaction to a ranked-based regression, with the behavioral adaptation index as the dependent variable. Note that the analysis of the MF and MB parameters deviated from the preregistration and is therefore considered exploratory (for the method and results of the originally planned analysis as well as details on the deviations from it, see supplementary materials).

### Supplementary Information

ESM 1 (PDF 1.34 mb)

## Data Availability

Data and materials are available through the GitHub repository: https://github.com/ranigera/HabitApp.

## Code availability

Analysis and task codes are available through the GitHub repository: https://github.com/ranigera/HabitApp.

## Declarations

### Preregistration

This study was preregistered on the Open Science Framework (OSF): https://osf.io/3gr5p/. Any deviation from the planned analyses is reported in the text. In cases where we carried out additional analyses that tested similar hypotheses as other registered analyses, we considered them exploratory and explicitly stated that in the text.
