# Supplementary material for: A novel free-operant framework enables experimental habit induction in humans
Source: Behav Res Methods. 2023 Nov 21;56(4):3937–58. doi: 10.3758/s13428-023-02263-6 (PMC11133146; doi:10.3758/s13428-023-02263-6)
Supplement: Supplementary file 1 — (PDF 1.34 mb) [file 13428_2023_2263_MOESM1_ESM.pdf]

Supplementary materials for:

A novel free-operant framework enables experimental habit induction in humans

Rani Gera[1,2,3,4], Segev Barak[1,2,3] and Tom Schonberg[1,3]

Additional manipulation check: verifying that the reduction in responding following outcome devaluation was not due to the mere passage of time

In a secondary manipulation check, to account for the possibility that a reduction in entries following outcome devaluation may stem from the mere passage of time (which perhaps led to a general decline in engagement rates), we examined whether participants increased their entries on the post-devaluation day. To do so, we first calculated the square root of the relative proportion of entries following each manipulation (i.e., outcome devaluation, and the control manipulations on adjacent days) to create a measure that is equivalent to the individual behavioral adaptation index (see details in the Data analysis section in the main text). Comparing this index between outcome devaluation and the subsequent control manipulation using a paired t-test indicated that once the outcome had regained its value participants increased back their entries ($t132=9.06$, $p<0.001$; Supplementary Fig. 2). Furthermore, this was true in each of the experimental groups (all $ps<0.002$).

Verifying that the relationships found between sensitivity to outcome devaluation and self-initiated sessions' related measures (average daily sessions and density) hold when these measures do not include any manipulation day

As mentioned in the Methods section of the main text, to have more than only one "regular" training day (namely, with no manipulations) in the short training group, we included the control manipulation days in the calculations of the session indices. To make sure this had not affected the pattern of our results, we repeated the same analysis (rank-based regression; see Methods for details) on self-initiated session indices calculated after omitting all manipulation days. The pattern of the results remained the same as in the main analysis, with the average number of daily sessions main effect reaching statistical significance ($F_{1,125} = 5.09$, $p=0.026$).

Results for further hypotheses tested with the negative-binomial mixed-model regression of the main analysis

Using the main negative-binomial mixed-model with linear parameterization we tested whether training duration differentially affects recovery of response rate ("engagement reinstatement") after a temporary outcome devaluation. We hypothesized that the recovery might be lower following short training compared with extensive training. The simple interactions comparing the change in entries from the first control manipulation to the second (when the outcome regains its value) between the short training group and each of the extensive training group indicated no such effect was present ($ps>0.777$; Table 1).

Testing the relationship between individual differences in "behavioral monotonicity" and habit expression

We speculated that participants who engaged with the app in a similar manner across days "monotonic participants" would tend to be more habitual than non-monotonic participants. To

this end we used the data only of the (combined) extensive training group. We calculated the normalized variance (that is, variance-to-mean ratio) of the number of daily entries made by each participant (after omitting the devaluation day) and correlated this measure with the behavioral adaptation index. A one-sided Spearman correlation test did not yield a significant effect (Spearman r_{86}=0.09, p=0.203), leading us to reject our hypothesis.

Pre-registered analysis of model-based and model-free relationship with training duration and habit expression

Following previous work (Daw et al., 2011; Gillan et al., 2015), in our pre-registration we stated with regard to the two-step task we will extract the individual levels of model-based and model-free learning strategies using two different methods. We stated that we will: (1) Run a mixed-model logistic regression model with stay or switch in the first stage choice (coded as 1 and 0, respectively) as the dependent variable and the previous trial's reward (coded as 1 and -1, for reward or no reward, respectively), transition (coded as 1 and -1, for common or rare, respectively) and their interaction as the predictors. The intercept and slopes for each of the predictors for each participant were stated to be taken as random effects over subjects. The individual beta for reward for each participant was planned to be taken as the model-free index and the beta for the interaction of reward x transition as the model-based index. (2) Fit a full reinforcement learning computational model, similar to the one used by Sharp et al. (Sharp et al., 2016) (with respect to the participant-level part of the model). This model was planned to be used to estimate and extract the following parameters: reliance on model-based and model-free learning strategies at the first stage, reliance on model-free learning strategy on the second stage, perseverance and learning rate.

While we extracted the model-free and model-based indices using these two methods, we reported in our pre-registration that for the hypothesis testing we will use the model-free and

model-based indices extracted from the mixed-models logistic regression in a multiple linear regression where each of these indices and its interaction with group are independent variables and the behavioral adaptation index is the dependent variable. The model was structured as follows (in R lme4 syntax):

behavioral adaptation index ~ (model free index + model based index) * group

Where the group factor consists of the short training and the (combined) extensive training groups. This model did not yield any significant effect (ps>0.1333).

Note that for the perseverance and learning rate factors the pre-registered hypothesis tests was to extract these parameters from the computational model and enter each of them as an independent variable along with group and their interaction to a ranked-based regression with the behavioral adaptation index as the dependent variable (See in the main text), similar to the analysis of the baseline engagement rates. We retrospectively realized that this analysis is also more suitable for the model-based and model-free parameters because the computational model is expected to capture these indices in a more comprehensive (trial by trial) manner and the rank-based correlation is more suitable for the behavioral adaptation index. We therefore performed this analysis but consider it exploratory (see in the main text).

Reinforcement learning model

We formulated our computational model for the two-step task data as a variation of the model used by Daw et al. (Daw et al., 2011) with similar adaptations to those implemented by Sharp et al. (Sharp et al., 2016) with respect to the within-participant-level part of the model. We used the mixture model approach by which each participant uses a combination of model-free and model-based algorithms to learn state-action value functions Q(s,a) that maps each action

within each state to its expected future reward (as the basis of making choices during the task). The task includes three states in total: a first stage s_A which can lead to one of two second states s_B and s_C . Each stage includes two distinct possible actions a_A and a_B. On each trial t participants start at the first stage $s_{1,t}$ (always s_A) and choose one of two actions $a_{1,t}$. They are then transitioned to one of the two second stages $s_{2,t}$ (according to a pre-determined transition probability) and choose another action $a_{2,t}$. The second state action can result in winning or not winning a reward r_t.

Participant-level modelling

Model-free component

The model-free component in our model relies on adjustments introduced by Sharp et al. (Sharp et al., 2016) to previous work. The algorithm uses the following update rule for each trial t and stage i:

$$Q_{MF}(s_{i,t+1}, a_{i,t+1}) = (1 - \alpha) \cdot Q_{MF}(s_{i,t}, a_{i,t}) + r_t$$

where α is the learning rate parameter. The eligibility trace was fixed to 1 for each trial, resulting in full reward value propagation back to the first stage. All state-action pairs that were not chosen or visited on a given trial were discounted by $(1 - \alpha)$.

Model-based component

The model-based component in our model maps which second state is primarily linked with which first stage action by counting all transitions until trial t and calculating which of the two potential mappings is more likely. Then the algorithm uses the stage two updated action values with the following update rule:

$$Q_{MB}(s_1, a_1) = Argmax_a Q_{MF}(s_2, a_2)$$

Where s_2 is the second stage state that is mapped to $Q_{MB}(s_1, a_1)$.

Choice rule

To relate the model-free and model-based action values with behavior (choices) we used a SoftMax function for each stage. On the first stage, the probability of choosing an action was formulated as follows:

$$P(a_{1,t} = a|s_{1,t}) = \frac{\exp\left[\beta_{MB} \cdot Q_{MB}(s_{1,t}, a) + \beta_{MF} \cdot Q_{MF}(s_{1,t}, a) + p \cdot rep(a)\right]}{\Sigma_{a'} \exp\left[\beta_{MB} \cdot Q_{MB}(s_{1,t}, a') + \beta_{MF} \cdot Q_{MF}(s_{1,t}, a') + p \cdot rep(a')\right]}$$

β_{MB} and β_{MF} are the first stage inverse temperature parameters that determines the weight of the model-based and model-free action values, respectively. rep(a) is an indicator function that is set to 1 when the chosen action is the same as in the previous trial and 0 if it is not. p is the perseveration parameter (Lau & Glimcher, 2005).

For the second stage choice probability we used:

$$P(a_{2,t} = a|s_{2,t}) = \frac{\exp\left[\beta_2 \cdot Q_{MF}(s_{2,t}, a)\right]}{\Sigma_{a'} \exp\left[\beta_2 \cdot Q_{MF}(s_{2,t}, a')\right]}$$

β_2 is the second stage inverse temperature parameter.

<u>Group-level modelling</u>

We embedded the single participant data within a group-level model. All five parameters we described ($\beta_{MB}, \beta_{MF}, \beta_2, \alpha, p$) were taken as random effects, that is, were drawn separately for each participant from a group-level common distribution. We used a non-centered parameterization (Betancourt & Girolami, 2015) for which we estimated a mean, scale and individual error estimates for each parameter. We specified all parameters as being drawn from a normal distribution. For α we then used inverse logit transform to constrain its values to stay in the range [0,1]. All inverse temperature parameters ($\beta_{MB}, \beta_{MF}, \beta_2$) and all scales were forced to be positive. We used the following priors:

1) For the means of the inverse temperature parameters we used ~ N(0,5) and for their scales ~ half-Cauchy(0,2). We forced the means and scales to be positive.
2) For the means of α and p we used ~ N(0,2.5) and for their scales ~ half-Cauchy(0,1).
3) For the individual error parameters we used ~ N(0,1).

The computational model parameters were estimated through the No-U-Turn sampler based Hamiltonian Monte Carlo as implemented in the Stan (Stan Development Team, 2021) Bayesian inference engine. We used Stan through the RStan package (Team, 2018) in R to run our model. We ran four chains of 4000 samples. Then, we discarded the first 2,000 samples (warmup samples), visually verified that all chains converged and made sure that all $\hat{R}s$ were smaller than 1.01 (Gelman & Rubin, 1992).

Details about spotting cheaters

While conducting standard examination of the raw data we spotted a participant with an extreme number of entries without performing the first press of the sequence (585 times, almost 5 times greater than the next on the list). We then further examined the entry data of this participant and observed prolonged periods in which the intervals between entries were extremely similar (within the scope of milliseconds). To test this empirically, we went over the entire data across all the participants and conducted a few tests that proved beyond any doubt that this participant cheated. For example, we iteratively extracted blocks of 10 consecutive inter-entry intervals and tested if their standard deviation was less 50ms. This occurred 99 times in the suspected participant's data and not even a single time in any other participant. In order to cheat this way, to the best of our understanding, significant web development skills are required. It is most likely that the participant (or someone else on their behalf) connected the

smartphone to a computer and programmatically set a repetitive page refreshing through some system with developer tools. Besides this participant, we did not spot any additional abnormalities in the data and we assume all other data was created in a reliable manner.

A remark about the app entries recordings

One thing we technically were not able to control was the initiation of an entry in the specific case where the smartphone is "awakened" by the user after it had shifted to sleep mode (by the user or following a preset period with no phone use) when the app was open. Nevertheless, we still had some control in that that participants had to enter the sequence in order to search for gold or to simply exit the app. It is very unlikely that this has affected the results because: (1) we assume this was uncommon. (2) There were hardly any "trials" in which participants did not complete the sequence. (3) Participants tend to enter a few times in row (self-initiated sessions) and thus get accustomed to exit the app as part of each "trial", which reduces the likelihood of the phone getting into sleep mode while the app is open on the screen. (4) It is very unlikely that the number of such occurrences would be different between groups.

A remark about the behavioral adaptation index

In the special case where the values across all three main manipulation days were zeros (occurred in one participant) we manually set the behavioral adaptation index to zero (since it cannot be explicitly calculated due to a division by 0).

Supplementary figures

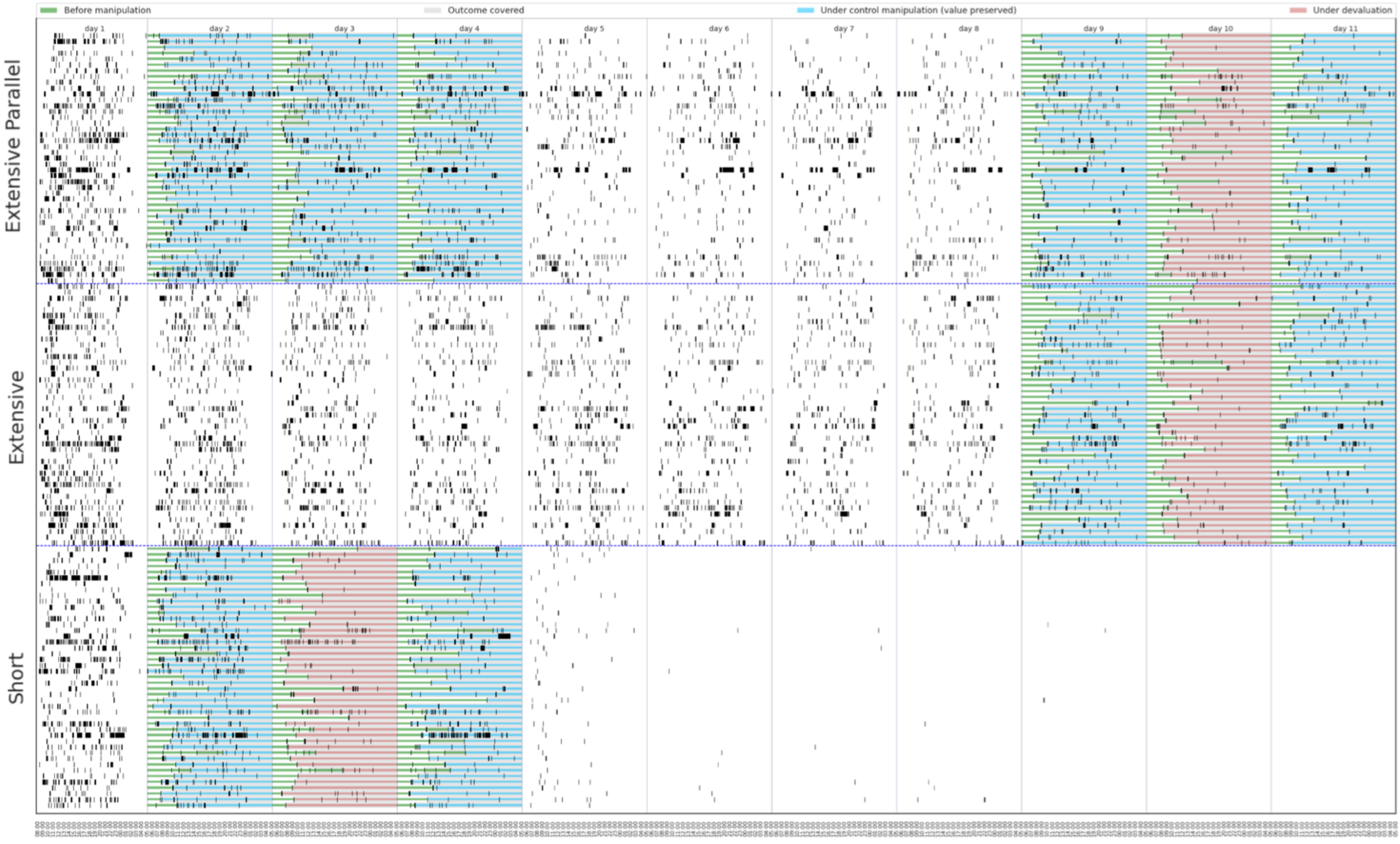

Supplementary Fig. 1. Participants entries throughout the task. A raster plot depicting participants' entries (each vertical line represents an entry) over the entire participation period (the short training group's period spanned four days).

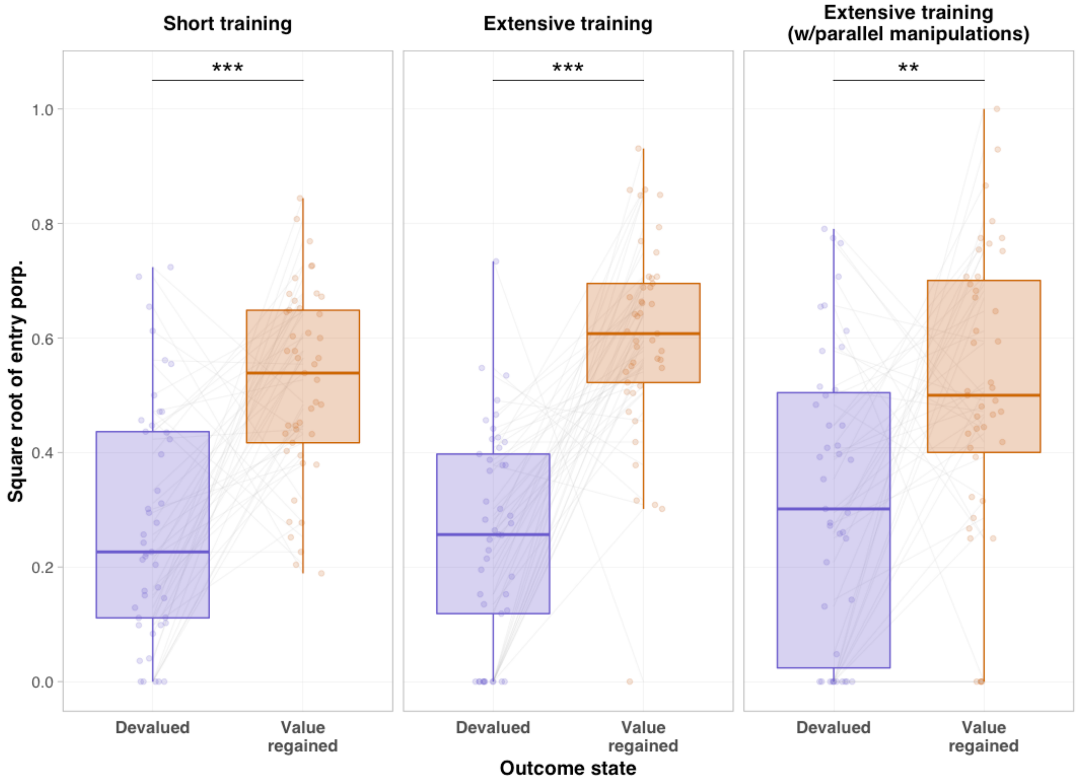

Supplementary Fig. 2. Entry change following temporal outcome devaluation. Participants' square root of entry proportion following outcome devaluation and a day later (where the outcome regained its value) following control manipulation. The proportions where first calculated on all three main manipulation days (including the pre-devaluation day) before the square root was taken. This index was chosen as it is equivalent to behavioral adaptation index (see in the main text).

Supplementary tables

Table S1

	Entries		
Predictors	*Log-Mean*	*std. Error*	*p*
(Intercept)	3.77	0.21	**<0.001**
Manipulation [Devaluation]	-2.02	0.21	**<0.001**
Manipulation [Control - post]	-0.64	0.19	**0.001**
Group [Extensive Training]	-0.50	0.29	0.086
Group [Extensive Training - Parallel week1 manipulations]	-1.18	0.30	**<0.001**
Manipulation [Devaluation] * Group [Extensive Training]	0.09	0.29	0.762
Manipulation [Control - post] * Group [Extensive Training]	0.40	0.26	0.129
Manipulation [Devaluation] * Group [Extensive Training - Parallel week1 manipulations]	0.83	0.30	**0.006**
Manipulation [Control - post] * Group [Extensive Training - Parallel week1 manipulations]	0.19	0.28	0.482

Results from the unchosen mixed-model negative binomial regression analysis (with a "quadric parameterization" parameterization of the variance) of participant entries following manipulations as explained by Manipulation type, Group and their interactions. Control pre-devaluation manipulation and the short training group were used as reference levels.

Table S2

	Entries		
Predictors	*Log-Mean*	*std. Error*	*p*
(Intercept)	3.52	0.20	**<0.001**
Manipulation [Devaluation]	-2.30	0.21	**<0.001**
Manipulation [Control - post]	-0.78	0.20	**<0.001**
Group [Extensive Training]	-0.48	0.29	0.094
Group [Extensive Training - Parallel week1 manipulations]	-1.22	0.29	**<0.001**
Manipulation [Devaluation] * Group [Extensive Training]	0.25	0.30	0.416
Manipulation [Control - post] * Group [Extensive Training]	0.49	0.28	0.081
Manipulation [Devaluation] * Group [Extensive Training - Parallel week1 manipulations]	0.85	0.31	**0.006**
Manipulation [Control - post] * Group [Extensive Training - Parallel week1 manipulations]	0.30	0.29	0.305

Results from the unchosen observation-level random effects (OLRE) of participant entries following manipulations as explained by Manipulation type, Group and their interactions. Control pre-devaluation manipulation and the short training group were used as reference levels.

References

Betancourt, M., & Girolami, M. (2015). Hamiltonian Monte Carlo for Hierarchical Models. *Current Trends in Bayesian Methodology with Applications*, 79–102. https://doi.org/10.1201/B18502-11/HAMILTONIAN-MONTE-CARLO-HIERARCHICAL-MODELS-MICHAEL-BETANCOURT-MARK-GIROLAMI

Daw, N. D., Gershman, S. J., Seymour, B., Dayan, P., & Dolan, R. J. (2011). Model-based influences on humans' choices and striatal prediction errors. *Neuron*, *69*(6), 1204–1215. https://doi.org/10.1016/j.neuron.2011.02.027

Gelman, A., & Rubin, D. B. (1992). Inference from Iterative Simulation Using Multiple Sequences. *Statistical Science*, *7*(4), 457–472.

Gillan, C. M., Otto, A. R., Phelps, E. A., & Daw, N. D. (2015). Model-based learning protects against forming habits. *Cognitive, Affective and Behavioral Neuroscience*, *15*(3), 523–536. https://doi.org/10.3758/s13415-015-0347-6

Lau, B., & Glimcher, P. W. (2005). DYNAMIC RESPONSE-BY-RESPONSE MODELS OF MATCHING BEHAVIOR IN RHESUS MONKEYS. *Journal of the Experimental Analysis of Behavior*, *84*(3), 555–579. https://doi.org/10.1901/JEAB.2005.110-04

Sharp, M. E., Foerde, K., Daw, N. D., & Shohamy, D. (2016). Dopamine selectively remediates 'model-based' reward learning: a computational approach. *Brain*, *139*(2), 355–364. https://doi.org/10.1093/BRAIN/AWV347

Stan Development Team. (2021). *Stan modeling language users guide and reference manual. Version 2.21.0. URL: http://mc-stan.org/* (p. 379). http://mc-stan.org/

Team, S. D. (2018). *RStan: the R interface to Stan*. http://mc-stan.org/
